# A Comprehensive Review of Swarm Optimization Algorithms

**DOI:** 10.1371/journal.pone.0122827

**Published:** 2015-05-18

**Authors:** Mohd Nadhir Ab Wahab, Samia Nefti-Meziani, Adham Atyabi

**Affiliations:** 1 Autonomous System and Advanced Robotics Lab, School of Computing, Science and Engineering, University of Salford, Salford, United Kingdom; 2 School of Computer Science, Engineering and Mathematics, Flinders University of South Australia, Adelaide, Australia; Politehnica University of Bucharest, ROMANIA

## Abstract

Many swarm optimization algorithms have been introduced since the early 60’s, Evolutionary Programming to the most recent, Grey Wolf Optimization. All of these algorithms have demonstrated their potential to solve many optimization problems. This paper provides an in-depth survey of well-known optimization algorithms. Selected algorithms are briefly explained and compared with each other comprehensively through experiments conducted using thirty well-known benchmark functions. Their advantages and disadvantages are also discussed. A number of statistical tests are then carried out to determine the significant performances. The results indicate the overall advantage of Differential Evolution (DE) and is closely followed by Particle Swarm Optimization (PSO), compared with other considered approaches.

## Introduction

Swarm Intelligence (SI) has attracted interest from many researchers in various fields. Bonabeau defined SI as “*The emergent collective intelligence of groups of simple agents*” [1]. SI is the collective intelligence behaviour of self-organized and decentralized systems, e.g., artificial groups of simple agents. Examples of SI include the group foraging of social insects, cooperative transportation, nest-building of social insects, and collective sorting and clustering. Two fundamental concepts that are considered as necessary properties of SI are self-organization and division of labour. Self-organization is defined as the capability of a system to evolve its agents or components in to a suitable form without any external help. Bonabeau et al. [1] also stated that self-organization relies on four fundamental properties of positive feedback, negative feedback, fluctuations and multiple interactions. Positive and negative feedbacks are useful for amplification and stabilization respectively. Fluctuations meanwhile are useful for randomness. Multiple interactions occur when the swarms share information among themselves within their searching area. The second property of SI is division of labour which is defined as the simultaneous execution of various simple and feasible tasks by individuals. This division allows the swarm to tackle complex problems that require individuals to work together [1].

This paper outline starts with brief discussion on seven SI-based algorithms and is followed by general discussion on others available algorithms. After that, an experiment is conducted to measure the performance of the considered algorithms on thirty benchmark functions. The results are discussed comprehensively after that with statistical analysis in the following section. From there, the two best performing algorithms are selected to investigate their variants performance against the best performing algorithm in five benchmark functions. The conclusion section is presented at the end of this paper.

### Swarm Intelligence Algorithms

This section introduces several SI-based algorithms, highlighting their notable variants, their merits and demerits, and their applications. These algorithms include Genetic Algorithms (GA), Ant Colony Optimization (ACO), Particle Swarm Optimization (PSO), Differential Evolution (DE), Artificial Bee Colony (ABC), Glowworm Swarm Optimization (GSO), and Cuckoo Search Algorithm (CSA).

### Genetic Algorithm

The Genetic Algorithm (GA) introduced by John Holland in 1975 [2, 3], is a search optimization algorithm based on the mechanics of the natural selection process. The basic concept of this algorithm is to mimic the concept of the ‘survival of the fittest’; it simulates the processes observed in a natural system where the strong tends to adapt and survive while the weak tends to perish. GA is a population based approach in which members of the population are ranked based on their solutions’ fitness. In GA, a new population is formed using specific genetic operators such as crossover, reproduction, and mutation [4–7]. Population can be represented in a set of strings (referred to as chromosomes). In each generation, a new chromosome (a member of the population) is created using information originated from the fittest chromosomes of the previous population [4–6]. GA generates an initial population of feasible solutions and recombines them in a way to guide their search toward more promising areas of the search space. Each of these feasible solutions is encoded as a chromosome, also referred to as genotype, and each of these chromosomes will get a measure of fitness through a fitness function (evaluation or objective function). The value of fitness function of a chromosome determines its capability to endure and produce offspring. The high fitness value indicates the better solution for maximization and the low fitness value shows the better solution for minimization problems. A basic GA has five main components: a random number generator, a fitness evaluation unit, a reproduction process, a crossover process, and a mutation operation. Reproduction selects the fittest candidates of the population, while crossover is the procedure of combining the fittest chromosomes and passing superior genes to the next generation, and mutation alters some of the genes in a chromosome [4–7].

**Fig 1:**
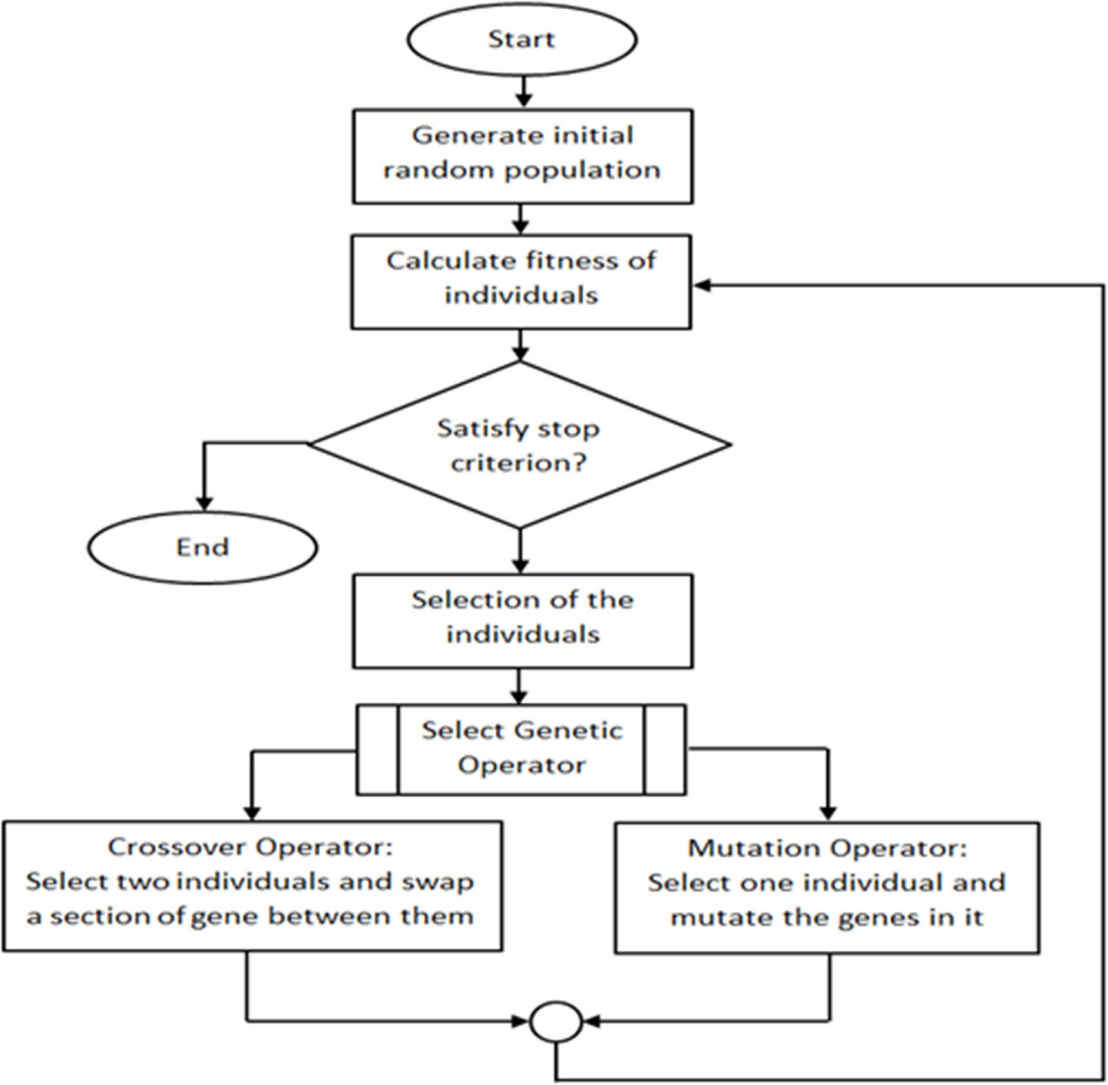
Flow Chart of Genetic Algorithm with all steps involved from beginning until termination conditions met [6].

Fig 1 shows the general flow chart of GA and the main components that contribute to the overall algorithm. The operation of the GA starts with determining an initial population whether randomly or by the use of some heuristics. The fitness function is used to evaluate the members of the population and then they are ranked based on the performances. Once all the members of the population have been evaluated, the lower rank chromosomes are omitted and the remaining populations are used for reproduction. This is one of the most common approaches used for GA. Another possible selection scheme is to use pseudo-random selection, allowing lower rank chromosomes to have a chance to be selected for reproduction. The crossover step randomly selects two members of the remaining population (the fittest chromosomes) and exchanges and mates them. The final step of GA is mutation. In this step, the mutation operator randomly mutates on a gene of a chromosome. Mutation is a crucial step in GA since it ensures that every region of the problem space can be reached. Elitism is used to prevent the best solution of the population from being destroyed during crossover and mutation operation. Elitism guarantees the fitness of new generation will be at least as good as current generation. The evaluation and generation of the new populations continue until the maximum number of generations is reached or the optimum solution is found. GA is advantageous in terms of requiring limited parameter settings and initialising itself from possible solutions rather than a single solution. One of the main drawbacks of GA is the lack of fast convergence towards the optimal values since the crossover and mutation process are random [6, 7]. The applications of GA are wide ranging from scheduling [8, 9], machine learning [10], robotics [11, 12], signal processing [13], business [14], mathematics [15], manufacturing [16, 17], routing [18], and many more.

Since the introduction of GA, many researchers have conducted studies to improve the performance of the GA. They have introduced several alternative approaches for crossover and mutation to enhance the quality of solutions. In crossover, instead of selecting one crossover point, De Jong *et al*. (1992) and Üçoluk (2002) have introduced N-point crossover and segmented crossover which selects several points for crossover [19, 20]. The difference between them is N-point crossover is choosing several breaking points randomly, while in segmented crossover, only two breaking points are utilized. Mutation is one of the most important operators in GA in order to direct the chromosomes towards the better solution. Therefore, several studies have given different methods for mutation. By default, each gene in a chromosome is assigned with probability, *p*_*m*,_ and mutated depending on that probability. This mutation is known as uniform mutation. The other approaches for mutation are bitwise inversion where the whole gene in a chromosome is mutated using a random mutation [19]. *Adaptive genetic algorithms* have been introduced in order to allow the use of precise parameters in setting the population size, the crossing over probability, and the mutation probability. All of these parameters are dynamic and changing over the iterations. For instance, if the population is not improving, the mutation rate is increasing and whenever the population is improving, the mutation rate starts decreasing [21]. Raja and Bhaskaran [22] have suggested a new approach of GA initialization that improve the overall performance of GA. In this approach, they utilized initialization twice where the first initialization is uses to identify the promising area. After the first initialization, all chromosome are ranked and the best chromosomes are selected. After that, GA is initialize again within the area where the best chromosomes have been identified.

### Ant Colony Optimization

**Fig 2:**
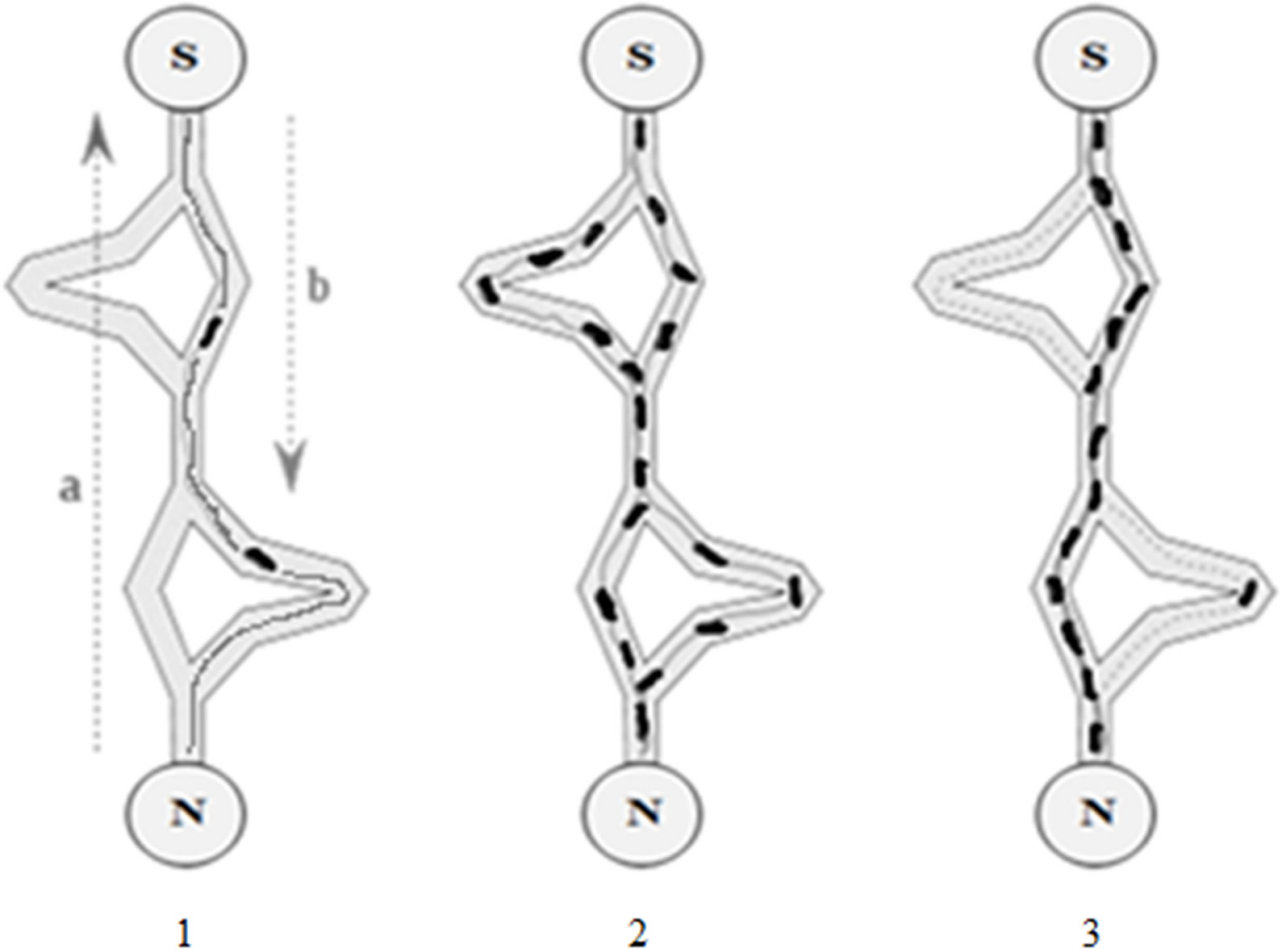
Ant Colony Optimization Algorithm processes. N and S denote Nest and Source with *a* is ongoing direction and *b* is returning direction. Sub Figure 2.1 shows early process where ants start find a path between nest and source and lay pheromone. Figure 2.2 shows intermediate process where ants went through all possible paths. Figure 2.3 shows most of ants choose path with highest pheromone [18].

**Fig 3:**
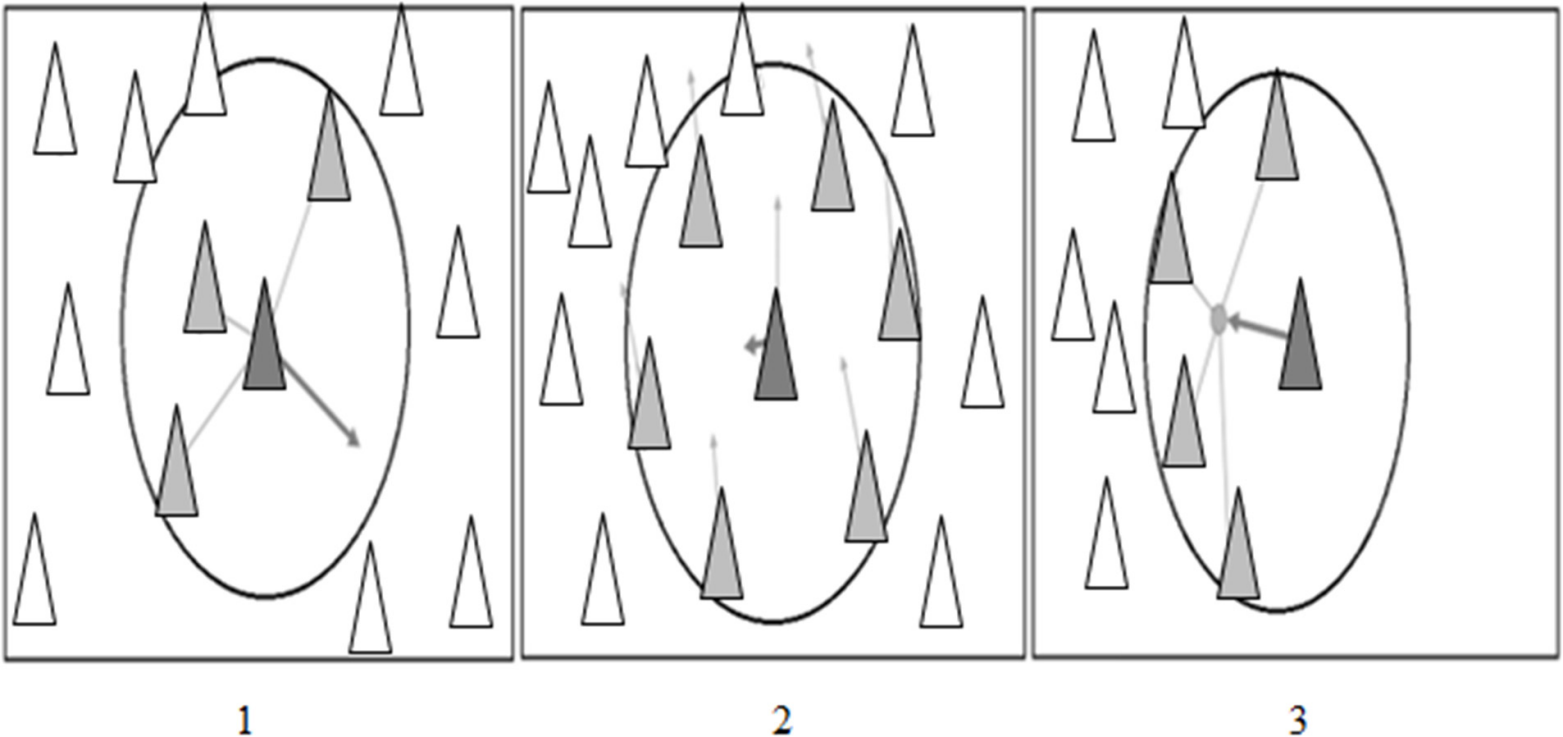
PSO Basic Behaviors. Figure 3.1 shows separation behavior where particle avoiding other particles. Figure 3.2 shows alignment behavior where particle moving towards head of local flockmates and maintain the speed between them. Figure 3.2 shows cohesion behavior where particle moving towards the average position of local flockmates [30].

Ant Colony Optimization (ACO) is a metaheuristic approach inspired by the Ant System (AS) proposed by Marco Dorigo in 1992 in his PhD thesis [23–25]. It is inspired by the foraging behaviour of real ants. This algorithm consists of four main components (ant, pheromone, daemon action, and decentralized control) that contribute to the overall system. Ants are imaginary agents that are used in order to mimic the exploration and exploitation of the search space. In real life pheromone is a chemical material spread by ants over the path they travel and its intensity changes over time due to evaporation. In ACO the ants drop pheromones when traveling in the search space and the quantities of these pheromones indicate the intensity of the trail. The ants choose the direction based on path marked by the high intensity of the trail. The intensity of the trail can be considered as a global memory of the system. Daemon actions is used to gather global information which cannot be done by a single ant and uses the information to determine whether it is necessary to add extra pheromone in order to help the convergence. The decentralized control is used in order to make the algorithm robust and flexible within a dynamic environment. The importance of having a decentralized system in ACO is due to resulting flexibility in the face of ant lost or ant failure offered by such a system. These basic components contribute to a cooperative interaction that leads to the emergence of shortest paths [23, 24]. Fig 2.1, 2.2, and 2.3 depict the initial phase, mid-range status of any system, and the final outcomes of the ACO algorithm respectively. The left figure illustrates the initial environment when the algorithm starts, where an agent (ant) starts moving randomly from the nest towards the source and returns back. The middle figure illustrates several iterations of execution when ants discover multiple possible paths between nest and source. The shortest path is chosen, and ants use this path frequently which contributes to high intensity of pheromone trail as shown in the sub-figure 3 in Fig 2. *N*, *S*, *a*, and *b* represent nest, food source, on-going path, and returning path respectively. The steps involved to find the best solution starts with choosing the next node (from the current position in the search space) using following equation:

$$p_{\left(i,j\right)}^{k}(t)=\frac{\left({\left[\tau_{ij}(t)\right]}^{\alpha }\cdot {\left[\eta_{ij}\right]}^{\beta }\right)}{\left(\sum_{k\in J_{k}}{\left[\tau_{ij}(t)\right]}^{\alpha }\cdot {\left[\eta_{ij}\right]}^{\beta }\right)}$$ (1)

*p*_*i*,*j*_ is the probability of going from node *i* to node *j*. *J*_*k*_ are the nodes that the ant is allowed to travel to from node *i*. *η*_*i j*_ contributes to the visibility between node *i* and node *j*. *τ*_*ij*_(*t*) represents the amount of un-evaporated pheromone between node *i* and node *j* at time *t*. α and β in Eq 1 control the influence of τij(t) and ηi j, where if alfa is higher, the searching behaviour of ant is more depending on pheromone and if beta is higher, the searching behaviour of ant is depending on its visibility or knowledge. Each ant also has a taboo list which is used to prevent any ants from visiting the same node twice.

Pheromones, as stated before, are one of the crucial components in ACO as they leave trails which increase the probability of the next ant choosing the same path. In order to deposit a pheromone, the following equation is used:

$$\Delta \tau_{ij}^{k}\left(t\right)=\{\begin{array}{c} \frac{Q}{L_{k}}\left(t\right)\\ 0 \end{array}$$ (2)

*Q* is a constant, *L* is the cost of the ant's tour, (i.e., the length of the generated path), *t* is the iteration number and *k* represents a specific ant. The value represents the pheromone rate between node *i* and node *j* that the ant visited in iteration *t*. The pheromone deposition value for a path that is not selected is zero. Another important component is the pheromone evaporation rate. This component determines the exploration and exploitation behaviour of the ant. High and low evaporation rates result in exploration and exploitation behaviours respectively. Too high exploration rates result in ants getting lost, while too low values result in an inability to acquire the optimal path [23, 24]. The pheromone decay factor is utilized using following equation:

$$\tau_{\left(i,j\right)}(t+1)=(1-p)\cdot \tau_{\left(i,j)(t\right)}+\sum_{\left(k=1\right)}^{m}\left[\Delta \tau_{\left(i,j\right)}^{k}(t)\right]$$ (3)

*m* is the number of ants in the system and *p* is the pheromone evaporation rate or decay factor. ACO has several advantages over other evolutionary approaches including offering positive feedback resulting in rapid solution finding, and having distributed computation which avoids premature convergence. These are in addition to taking advantage of the existing collective interaction of a population of agents [26, 27]. However, ACO has drawbacks such as slower convergence compared with other heuristic-based methods and lack a centralized processor to guide it towards good solutions. Although the convergence is guaranteed, the time for convergence is uncertain. Another important demerit of ACO is its poor performance within problems with large search spaces [26, 27]. ACO has been applied in various optimization problems such as traveling salesman problem (TSP) [28], quadratic assignment problem [29], vehicle routing [30], network model problem [31, 32], image processing [33], path planning for mobile robot [34], path optimization for UAV System [35], project management [36] and so on.

A number of ACO variants have been created with the aim to improve overall performance. Two years after the introduction of ACO, Dorigo and Gambardella made modifications by improving three major aspects (pheromone, state transition rule and local search procedures) which produce the variant of ACO called Ant Colony System (ACS) [37]. GA is initialize again

ACS uses centralise (global) update approach for pheromone update and only concentrate the search within a neighbourhood of the best solution found so far in order to increase efficiency for convergence time. The state transition rule is different from ACO where ACS has a stated probability (*q*_*0*_) to decide which behaviour is used by the ant. *q*_*0*_ is usually set to 0.9 and compare to a value of *q* (which 0 ≤ *q* ≤ 1). If the value of *q* is less than that, then exploitation behaviour is used and vice versa. For local search procedures, a local optimization heuristic based on an edge exchange strategy such as 2-opt, 3-opt or Lin-Kernighan is applied to each solution generated by an ant to get its local minima. This combination of new pheromone management, new state transition, and local search procedures has produced a variant of ACO for TSP problems [37]. Max-Min Ant System (MMAS) is considered as another notable variant of ACO. The approach was introduced by Stutzle and Hoos in 2000 and it limits the pheromone trail values within the interval of [τ_min_, τ_max_] [38]. MMAS also modified three aspects of ACO. First, at the beginning, the pheromone trails are set to the maximum value which escalate the exploration behaviour of the ants. Second, the authors introduce an interval of [τ_min_, τ_max_] which limits the pheromone trails in order to avoid stagnation. Third, only one ant is allowed to add pheromone which help exploiting the best solutions found during the execution of the algorithm. The pheromone may be added by using either an *iteration-best* approach or a *global-best* approach. In the *iteration-best* approach, only the ant with best solution adds the pheromone for each iteration while in the *global-best* approach, the ant with the best solution can add the pheromone without considering other ants in the same iteration [38].

### Particle Swarm Optimization

Particle Swarm Optimization (PSO) is an optimization technique introduced by Kennedy and Eberhart in 1995 [39]. It uses a simple mechanism that mimics swarm behaviour in birds flocking and fish schooling to guide the particles to search for global optimal solutions. Del Valle and his co-authors [40] described PSO with three simple behaviours of separation, alignment, and cohesion as shown in Fig 3 respectively. Separation is the behaviour of avoiding the crowded local flockmates while alignment is the behaviour of moving towards the average direction of local flockmates. Cohesion is the behaviour of moving towards the average position of local flockmates. The formulas of PSO algorithm are as follows [39, 41]:

$$v_{id}^{t+1}=v_{id}^{t}+c_{1}\cdot rand(0,1)\cdot \left(p_{id}^{t}-x_{id}^{t}\right)+c_{2}\cdot rand(0,1)\cdot \left(p_{gd}^{t}-x_{id}^{t}\right)$$ (4)

$$x_{id}^{t+1}=x_{id}^{t}+v_{id}^{t+1}$$ (5)

where $v_{id}^{t}$ and $x_{id}^{t}$ are particle velocity and particle position respectively. *d* is the dimension in the search space, *i* is the particle index, and *t* is the iteration number. *c*_*1*_ and *c*_*2*_ represent the speed, regulating the length when flying towards the most optimal particles of the whole swarm and the most optimal individual particle. *p*_*i*_ is the best position achieved so far by particle *i* and *p*_*g*_ is the best position found by the neighbours of particle *i*. *rand*(0,1) is the random values between 0 and 1. The exploration happens if either or both of the differences between the particle’s best ($p_{id}^{t}$) and previous particle’s position ($x_{id}^{t}$), and between population’s all-time best ($p_{gd}^{t}$) and previous particle’s position ($x_{id}^{t}$) are large, and exploitation occurs when these values are both small. PSO proved to be an efficient optimization algorithm by searching an entire high-dimensional problem space. It is a robust stochastic optimization technique based on the movement and intelligence of swarms. It applies the concept of social interaction to problem solving and does not use the gradient of the problem being optimized, so it does not require the optimization problem to be differential, as is required by classic optimization methods [42]. The optimization of irregular problems that are noisy and change over time can be determined using PSO [43–45]. The parameters of PSO consist of number of particles, position of agent in the solution space, velocity and neighbourhood of agents (communication of topology).

**Fig 4:**
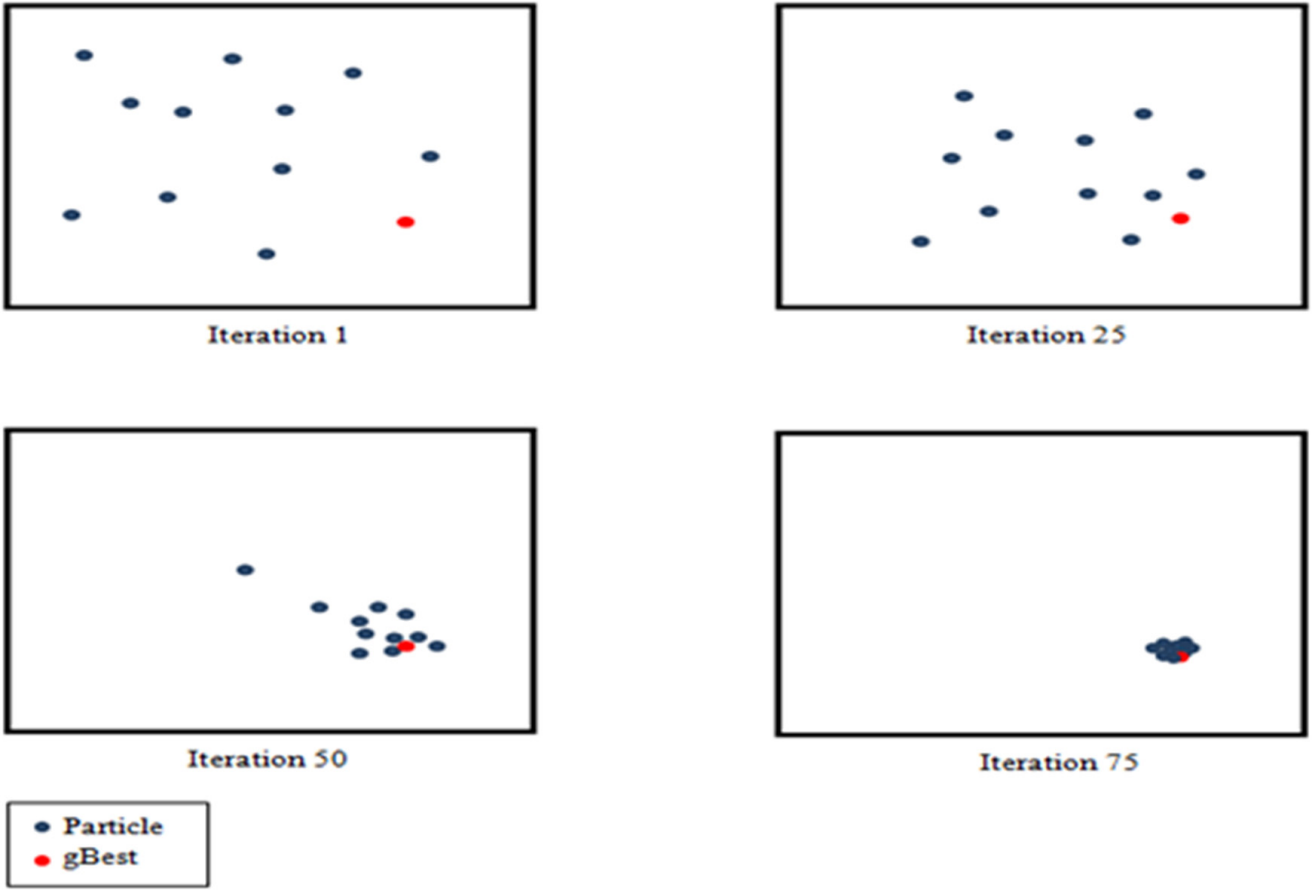
Particle Swarm Optimization movement towards global optima over iteration numbers [33].

The PSO algorithm begins by initializing the population first. The second step is calculating the fitness values of each particle, followed by updating individual and global bests, and later, the velocity and the position of the particles get updated. The second to fourth steps get repeated until the termination condition is satisfied [40, 46–48]. Fig 4 illustrates the PSO algorithm output over iterations. In the first iteration, all particles spread out in order to find the best solution (exploration). Each particle is evaluated. The best solutions are found with respect to neighbourhood topology and the personal and global best particles for each member of the swarm are updated. The convergence would be achieved through attracting all particles towards the particle with the best solution.

The PSO algorithm has many merits. It is simple to implement, has only a few parameters to be set, it is effective in global search, it is insensitive to scaling of design variables, and it is easily parallelized for concurrent processing [48–50]. PSO has tendency to result in a fast and premature convergence in mid optimum points, in addition to having slow convergence in a refined search area (having weak local search ability) [48–50]. PSO is used in networking [51], power systems [52], signal processing [53], control system [54], machine learning [55], image processing [56–58], and many more.

There are several approaches that can be used to improve PSO in general. The size of the population is one of the important factors. Higher population size can increase the chance of faster and precise convergence. A second approach is to achieve a balance between exploration and exploitation. In the beginning of iteration, high exploration would give a high chance to find a solution which is close to global optima. Meanwhile towards the end of iteration, high exploitation would give a chance for particle to find the most accurate solution within the promising area. A sub-swarm approach is another way that can be used to increase the basic PSO performance which is quite commonly used nowadays. Allocating different tasks or objectives to each sub-swarm can also increase the efficiency of PSO in the multi-objective problems [59]. Another approach to improve the PSO performance is to set the contributing components of the velocity equation (dynamic velocity adjustment). Such an approach can direct particles in different directions resulting in faster convergence towards a global optimum [60].

The two most notable variants in PSO are the introduction of inertia weight and constriction factors. Inertia weight (*w*) is introduced by Shi and Eberhart three years after PSO was first introduced to regulate the influence of previous velocity which also controls the exploration and the exploitation behaviours of particle [61]. If the *w* value is high then the step size is big, resulting in the occurrence of exploration behaviour. If the *w* value is low then the step size is small and the exploitation behaviour occurs. This element has been accepted as new standard form of velocity equation for basic PSO as illustrated in Eq (6):

$$v_{id}^{t+1}=w\cdot v_{id}^{t}+c_{1}\cdot rand(0,1)\cdot \left(p_{id}^{t}-x_{id}^{t}\right)+c_{2}\cdot rand(0,1)\cdot \left(p_{gd}^{t}-x_{id}^{t}\right)$$ (6)

The introduction of inertia weight has improved overall performance of PSO in terms of the speed of convergence and the quality of solutions. From there, much research has been done to find the best configuration for inertia weight in order to optimize the convergence speed and the solutions’ quality. Bratton and Kennedy suggested to use an inertia weight value higher than 1.0 and decreasing eventually to a value lower than 1.0 with the aim of encouraging exploration at an early stage and exploitation of the best area found towards the end [62]. Clerc and Kennedy later introduced the constriction factor named as *K* in order to increase the chance of convergence and avoid particles from leaving the search space [63].

$$v_{id}^{t+1}=K[v_{id}^{t}+c_{1}\cdot rand(0,1)\cdot \left(p_{id}^{t}-x_{id}^{t}\right)+c_{2}\cdot rand(0,1)\cdot \left(p_{gd}^{t}-x_{id}^{t}\right)]$$ (7)

Both variants have improved the overall performance of the PSO algorithm. Eberhart and Shi have compared these two variants and come to the conclusion that the constricted PSO perform better than the improved basic PSO [64]. There are several elements in PSO such as swarm communication topology, and the number of particles which can determine the quality of the solution. Figueirdo and Ludermir have evaluated five types of communication topologies of global, local, von neuman, wheel and four clusters. They concluded that global topology shows promising results compared to other topologies [65]. Bratton and Kennedy investigated the effect of number of particles in finding the solutions. Their study showed that there is no absolute number of population size that can be applied for all optimization problems [62].

### Differential Evolution

The Differential Evolution (DE) algorithm is a population-based algorithm that can be considered to be similar to GA since it employs similar operators; crossover, mutation, and selection. The main difference between DE and GA is in constructing better solutions, where DE relies on mutation operation while GA relies on crossover operation. This algorithm was introduced by Storn and Price in 1997 [66]. Since this algorithm relies on mutation operation, it utilizes the mutation as a search mechanism and takes advantage of the selection operation in order to direct the search towards the potential regions in the search space. Target Vector, Mutant Vector, and Trail Vector are three properties that DE utilizes for generating a new population iteratively. The target vector is the vector that contains the solution for the search space; the mutant vector is the mutation of the target vector; and the trail vector is the resultant vector after the crossover operation between target vector and mutant vector. The basic steps of the DE algorithm as stated before, are similar to GA with only slight differences [67, 68]. DE starts with steps such as population initialization followed by evaluation to determine the fittest members of the population. Later, new parameter vectors get generated by adding the weighted difference of the two population vectors with the third vector. This step is referred to as mutation. Within the crossover, the vector is mixed and the algorithm takes a final step of selection. In order to see the differences between DE and GA, a more detailed discussion on the three main operators in DE is required.

**Fig 5:**
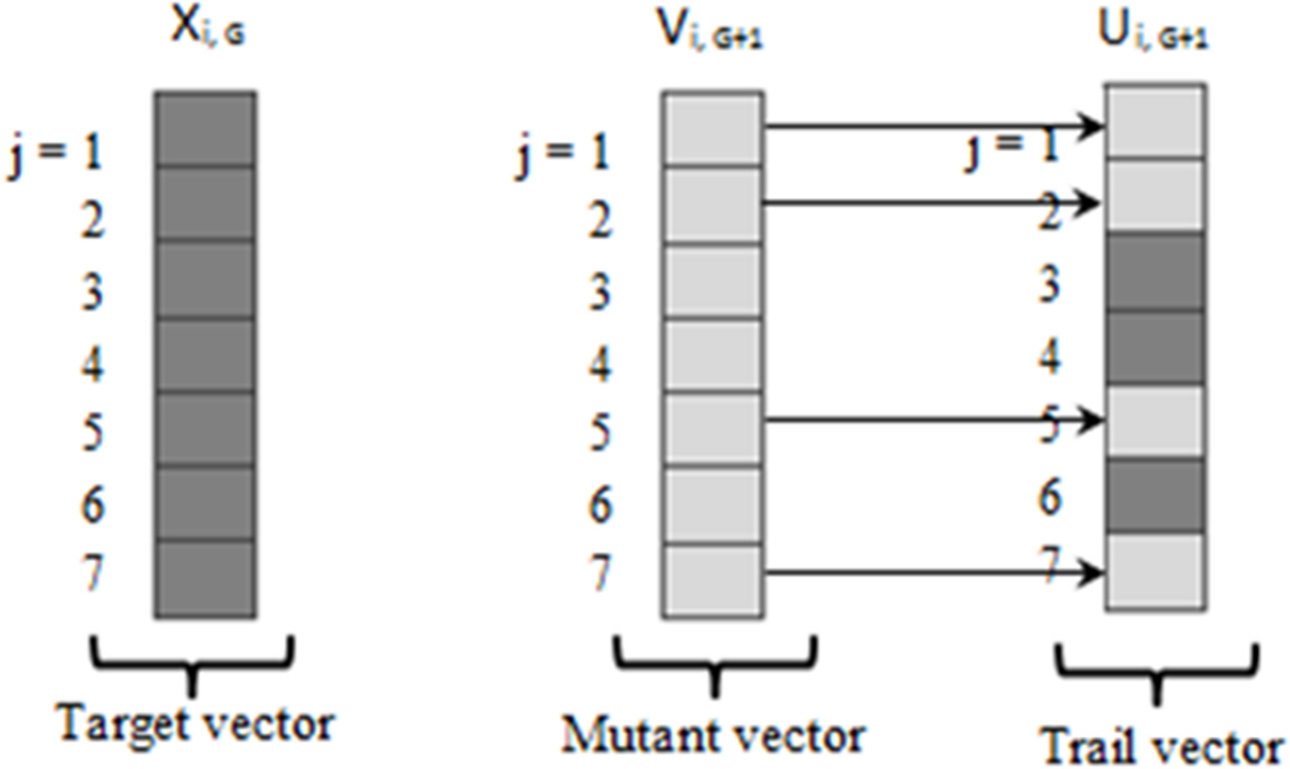
Illustration of Crossover Process of DE with vector dimension (*j*) of 7. Target vector is current solution with mutant vector is another possible solution. Trail vector is new solution after crossover process between target vector and mutant vector [43].

In the mutation step each of *N* parameter vectors goes through mutation. Mutation is the operation of expanding the search space and a mutant vector is generated by:

$$v_{i,G+1}=x_{r1,G}+F\left(x_{r2,G}-x_{r3,G}\right)$$ (8)

where *F* is the scaling factor with a value in the range of [0,1] with solution vectors *x*_*r*1,_ *x*_*r*2,_ and *x*_*r*3_ being chosen randomly and satisfying following criteria:

$$x_{r1},x_{r2},x_{r3}|r_{1}\neq r_{2}\neq r_{3}\neq i$$ (9)

where *i* is the index of the current solution. Fig 5 illustrates a two-dimensional vector which plays a part in generating the mutant vector.

Crossover operation is introduced to increase the diversity of the disconcerted parameter vectors. The parent vector is mixed with a mutated vector and a trial vector is produced by:

$$u_{i,G+1}=\{\begin{array}{c} v_{i,G+1}\;if\;R_{j}\leq CR\\ x_{i,G}\;if\;R_{j}>CR \end{array}$$ (10)

where *CR* is a crossover constant and *R*_*j*_ is a random real number between [0,1] with *j* denoting the *j* ^th^ component of the resultant array.

In DE, all solutions in the population have the same probability of being selected as parents without considering their fitness value. This is the main difference in the operations of DE and GA. Simply put, the child (trail vector) produced is only evaluated after mutation and crossover operations. After that, the performance of this child vector is compared to its parent and the better vector is retained in the population. The exploitation behaviour occurs when the difference between two solution vectors in Eq 6 are small, while the exploration behaviour occurs when the difference between those two are large. DE is advantageous in terms of enhancing the capacity of local search and keeping the multiplicity of the population while it suffers from slow convergence and being unstable [68]. DE is employed in various applications such as electrical engineering [69], image processing [70], machine learning [71], and economy [72].

In general, DE performance can be improved by increasing the population size. It can also balance between exploration and exploitation behaviour where the scaling factor (which determines the step size) is high at the beginning and decreases towards the end of an iteration. Another step that can be used is the introduction of elitism which can avoid the best solution from being destroyed when the next generation is created. There are many variants of DE available since its introduction by Storn and Price. Mezura-Montes *et al*. have discussed several variants of DE and done a comparative study between them [73]. The variants discussed are *DE/rand/1/bin*, *DE/rand/1/exp*, *DE/best/1/bin*, *DE/best/1/exp*, *DE/current-to-best/1*, *DE/current-to-rand/1*, *DE/current-to-rand/1/bin*, and *DE/rand/2/dir*. The differences between them are in terms of individuals selected for mutation, the numbers of pairs of solutions selected and the type of recombination [74]. In the study the variants of DE are described in *DE/x/y/z* form where *x* represents a string denoting the base vector to be perturbed; for example *rand* means that vectors selected randomly to produce the mutation values and *best* means that the best vectors among population is selected to produce the mutation values. *y* is the number of vectors considered to generate a new vector and is represented in an integer form which indicate the number of pairs of solutions used to produce a new solution. *z* represents the type of crossover, for instance *bin* and *exp* (*bin* meaning binomial and *exp* meaning exponential). Meanwhile, *current-to-best* and *current-to-rand* are arithmetic recombination proposed by Price [75] to eliminate the binomial and exponential crossover operator with the rotation invariant.

### Artificial Bee Colony

Artificial Bee Colony (ABC) is one of the most recent swarm intelligence algorithms. It was proposed by Dervis Karaboga in 2005 [76]; in 2007, the performance of ABC was analysed [77] and it was concluded that ABC performs quite well compared with several other approaches. This algorithm is inspired by the intelligent behaviour of real honey bees in finding food sources, known as nectar, and the sharing of information about that food source among other bees in the nest. This algorithm is claimed to be as simple and easy to implement as PSO and DE [78]. In this approach, the artificial agents are defined and categorized into three types, the employed bee, the onlooker bee, and the scout bee. Each of these bees has different tasks assigned to them in order to complete the algorithm’s process. The employed bees focus on a food source and retain the locality of that food source in their memories. The number of employed bees is equal to the number of food sources since each employed bee is associated with one and only one food source. The onlooker bee receives the information of the food source from the employed bee in the hive. After that, one of the food sources is selected to gather the nectar. The scout bee is in charge of finding new food sources and the new nectar. The general process of ABC method and the details of each step are as follows [76–78]:

Step 1. Initialization Phase: All the vectors of the population of food source, $\vec{x_{l}}$, are initialized (*i =* 1…*SN*, where *SN* is population size) by scout bees and control parameters being set. Each $\vec{x_{l}}$ vector holds *n* variables, which is optimized, to minimize the objective function. The following equation is used for initialization phase:

$$x_{i}=l_{i}+rand\left(0,1\right)*\left(u_{i}-l_{i}\right)$$ (11)

where *l*_*i*_ and *u*_*i*_ respectively are the lower and upper bound parameters of *x*_*i*_.

Step 2.Employed Bees Phase: In this phase, the search for a new food source, $\vec{v_{l}}$, increases in order to have more nectar around the neighbourhood of the food source, $\vec{x_{l}}$. Once a neighbouring food source is found, its profitability or fitness is evaluated. The new neighbouring food source is defined by using following formula:

$$v_{i}=x_{i}+{Ø}_{i}\left(x_{i}-x_{j}\right)$$ (12)

where *x*_*j*_ is a random selected food source and Ø_*i*_ is a random number of [*-a*, *a*]. Once the new food source, *v*_*i*_, is produced its profitability is measured and a greedy selection is applied between $\vec{x_{l}}$ and $\vec{v_{l}}$. The exploration happens if the difference between *x*_*i*_ − *x*_*j*_ is large and the exploitation behaviour is when the difference is small. The fitness value of the solution, *fit*_*i*_($\vec{x_{l}}$), is determined using following equation:

$$fit_{i}(\vec{x_{i}})=\{\begin{array}{c} \frac{1}{1+f_{i}(\vec{x_{i}})}\;if\;f_{i}(\vec{x_{i}})\geq 0\\ 1+abs(f_{i}(\vec{x_{i}}))\;if\;f_{i}(\vec{x_{i}})<0 \end{array}$$ (13)

where $f_{i}\vec{(x_{l}})$ is the objective function value of solution ($\vec{x_{i}}$).

Step 3. Onlooker Bees Phase: Onlooker bees that are waiting in the hive choose their food sources depending on probability values measured using the fitness value and the information shared by employed bees. The probability value, *p*_*i*_, is measured using the following equation:

$$p_{i}=\frac{fit_{i}\left(\vec{x_{i}}\right)}{\sum_{i=1}^{SN}fit_{i}\left(\vec{x_{i}}\right)}$$ (14)

Step 4. Scout Bees Phase: The scout bees are those unemployed bees that choose their food sources randomly. Employed bees whose fitness values cannot be improved through predetermined number of iterations, called as *limit* or *abandonment criteria*, become the scout bees and all their food sources get abandoned.

Step 5. The best fitness value and the position associated to that value are memorized.

Step 6. Termination Checking Phase: If the termination condition is met, the programme terminates, otherwise the programme returns to Step 2 and repeats until the termination condition is met.

Advantages of ABC include being easy to implement, robust, and highly flexible. It is considered as highly flexible since only requires two control parameters of maximum cycle number and colony size. Therefore, adding and removing bee can be done without need to reinitialize the algorithm. It can be used in many optimization problems without any modification, and it requires fewer control parameters compared with other search techniques [77–80]. The disadvantages of ABC include the requirement of new fitness tests for the new parameters to improve performance, being quite slow when used in serial processing, and the need for a high amount of objective function evaluations [81]. ABC has been implemented in various fields including engineering design problems [82, 83], networking [84], business [85], electronics [86], scheduling [86] and image processing [86].

Although ABC algorithm was only been introduced less than ten years ago there are already quite number of variants of ABC available. One of the important ABC variant is Interactive ABC (IABC) designed to solve numerical optimization problems [87]. Bao and Zeng have introduced three selection strategies of food source by onlooker bees for ABC which form three variants called Rank Selection Strategies ABC (RABC), Tournament Selection ABC (TABC) and Disruptive Selection ABC (DABC) [88]. The main aim for all these variants is to upsurge the population diversity and avoid premature convergence. Bao and Zeng have tested these modified ABCs with the standard ABC and the results showed that these three selection strategies perform better search compared with the standard ABC [88].

### Glowworm Swarm Optimization

**Fig 6:**
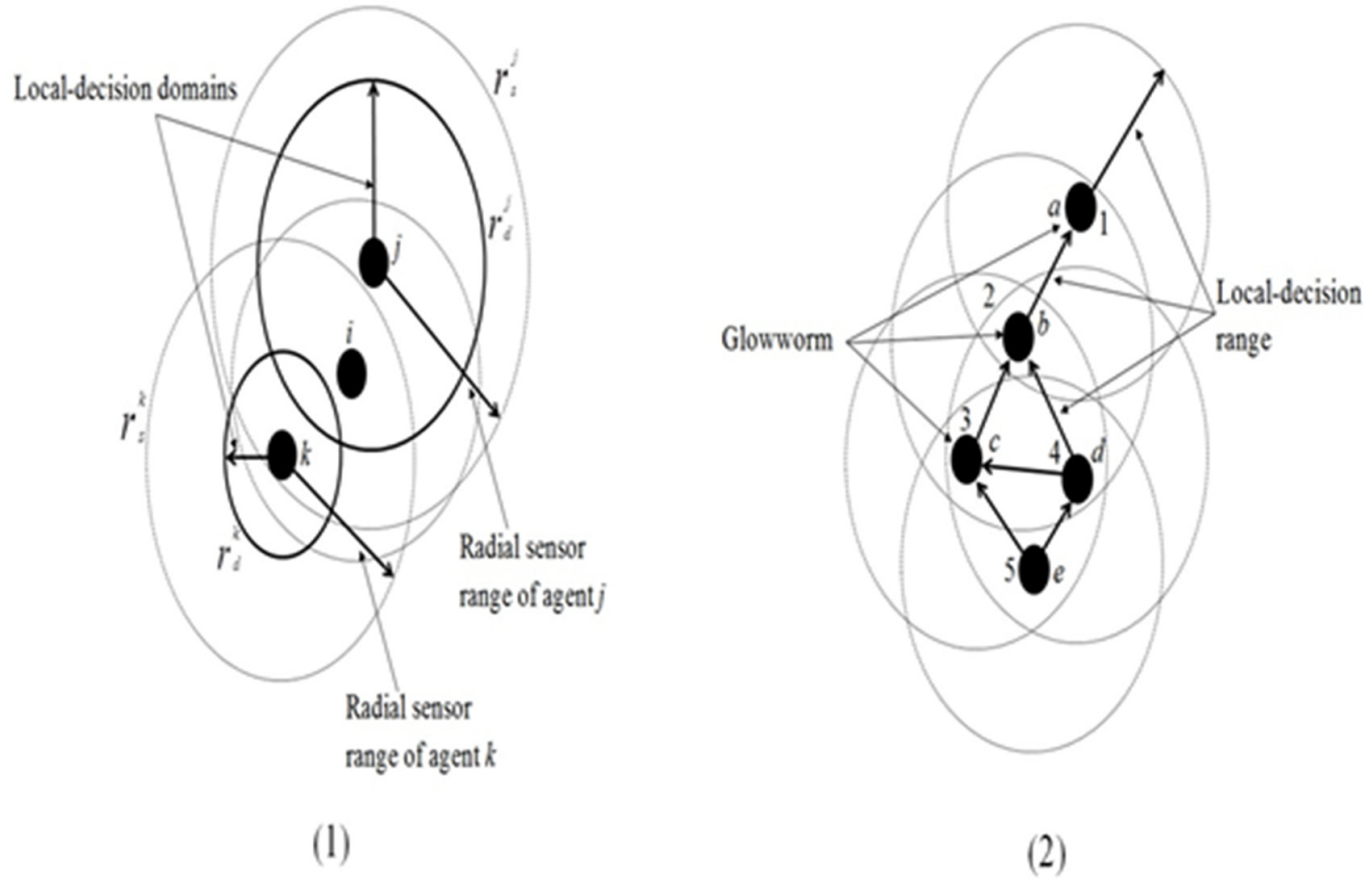
Glowworm Search Optimization (GSO) in two possible conditions. *a*, *b*, *c*, *d*, *e*, *f*, *i*, *j*, and *k* are the glowworm agents. In Figure 6.1, figure illustrates three glowworm agents with different sensor range and local-decision range. It shows if agent within local-decision of other agent, the agent with lower *luciferin* values move towards agent with higher *luciferin* values. In Figure 6.2, glowworm agents are ranked based on their *luciferin* values with lower number represent higher *luciferin* values and higher number represent lower *luciferin* values [58].

Glow worm Swarm Optimization (GSO) is a new SI-based technique aimed to optimize multi-modal functions, proposed by Krishnanad and Ghose in 2005 [89, 90]. GSO employs physical entities (agents) called glowworms. A condition of glowworm *m*, at time *t* has three main parameters of a position in the search space (*x*_*m*_(*t*)), a *luciferin* level (*l*_*m*_(*t*)) and a neighbourhood range (*r*_*m*_(*t*)). These three parameters change over time [89–91]. Initially the glowworms are distributed randomly in the workspace, instead of finite regions being randomly placed in the search area as demonstrated in ACO. Later, other parameters are initialized using predefined constants. Yet, similar to other methods, three phases are repeated until the termination condition is satisfied. These phases are *luciferin* level update, glowworm movement, and neighbourhood range update [89]. In order to update the *luciferin* level, the fitness of current position of a glowworm *m* is determined using following equation:

$$l_{m}\left(t\right)=\left(1-p\right)\cdot l_{m}\left(t-1\right)+\gamma J\left(x_{m}\left(t\right)\right)$$ (15)

where *p* is the *luciferin* evaporation factor, γ is the *luciferin* constant and *J* is an objective function. The position in the search space is updated using following equation:

$$x_{m}\left(t\right)=x_{m}\left(t-1\right)+s\left(\frac{x_{n}\left(t-1\right)-x_{m}\left(t-1\right)}{∥x_{n}\left(t-1\right)-x_{m}\left(t-1\right)∥}\right)$$ (16)

where *s* is the step size, and ||.|| is Euclidean norm operator. If the difference between *x*_*n*_ and *x*_*m*_ is large then exploration behaviour takes place and if this difference is small then exploitation behaviour occurs. Later, each glowworm tries to find its neighbours. In GSO, a glowworm *m* is the neighbour of glowworm *n* only if the distance between them is shorter than the neighbourhood range *r*_*m*_(*t*), and on condition where glowworm *n* is brighter than glowworm *m*. However, if a glowworm has multiple choices of neighbours, one neighbour is selected using the following probability equation.

$$p_{\text{m}}(\text{t})=\frac{l_{\text{m}}(\text{t})\text{-}l_{\text{n}}(\text{t})}{\sum_{\text{k}\in \text{Ni}(\text{t})}l_{\text{k}}(\text{t})\text{-}l_{\text{n}}(\text{t})}$$ (17)

where the probability of glowworm at *m* moving towards glowworm at *n* is the difference of *luciferin* level between them over difference of *luciferin* level between all glowworms within the range of glowworm *m*. The solution with the highest probability is selected and then the glowworm moves one step closer in direction of the chosen neighbour with a constant step size *s*. In the final phase, the neighbourhood range (*r*_*m*_(*t*)) is updated to limit the range of communication in a group of glowworms. The neighbourhood range is calculated using following equation:

$$r_{m}\left(t+1\right)=\text{min}\left\{r_{s},max\left[0,r_{m}\left(t\right)+\beta \left(n_{d}-\left|n_{m}\left(t\right)\right|\right)\right]\right\}$$ (18)

where *r*_*s*_ is a sensor range (a constant that limits the size of the neighbourhood range), *n*_*d*_ is the desired number of neighbours, |*n*_*m*_(*t*)| is a number of neighbours of the glowworm *m* at time *t* and *β* is a model constant. Fig 6 illustrates two possible circumstances in GSO’s agents’ evolving procedures in which with respect to agents’ positions in the search space and the available neighbouring agents different behaviours occurs. In (a), *i*, *j* and *k* represent the agents of glowworm. $r_{s}^{j}$ denotes the sensor range of agent *j* and $r_{d}^{j}$ denotes the local-decision range for agent *j*. The same applies with *i* and *k* where sensor range and local-decision range are represented by $r_{s}^{i}$ and $r_{d}^{i}$ and $r_{s}^{k}$ and $r_{d}^{k}$ respectively. It is applied in the circumstances where agent *i* is in the sensor range of agent *j* and *k*. Since the agents have different local-decision domains only agent *j* uses the information from agent *i*. In (b), *a*, *b*, *c*, *d*, *e*, and *f* are glowworm agents. 1, 2, 3, 4, 5, and 6 represent the ranking of the glowworm agents based on their *luciferin* values. Agents are ranked based on their *luciferin* values resulting in agent *a* being ranked 1 since it has the highest *luciferin* value. GSO is effective within applications with limited sensor range and is capable of detecting multiple sources and is applicable to numerical optimization tasks [89–91]. However, it also has low accuracy and slow convergence rate [92, 93]. GSO has been applied to routing [94], swarm robotics [95], image processing [96], and localization [97, 98] problems.

GSO can be improved in general by considering the following modifications. 1) Expanding the neighbourhood range to include all agents. Once the best solution has been determined, all agents can move towards the agent with the best solution. This step can increase the efficiency in exploitation, since higher number of agents to be within the best solution range. 2) In order to increase GSO’s convergence rate, the number of neighbours considered within the neighbourhood range need to be as small as possible. This step might reduce the time taken for GSO since less calculation required to determine the probability and direction of its movement.

GSO has several variants that improve the overall performance of GSO. For example, He *et al*. [99] introduced Improved GSO (IGSO) to take advantage of integrating chaos behaviour in order to avoid local optima and increasing the speed and accuracy of convergence. He *et al*. have tested their algorithm on six benchmark functions and the results showed IGSO outperform GSO [99]. Zhang *et al*. [100] have proposed two ideas to improve the performance of GSO. First, they proposed several approaches to alter the step-size of the glowworm such as fixed step, dynamic linear decreasing, and dynamic non-linear decreasing [100]. They have compared the variance of step-size approaches and the results showed that both dynamic linear and the non-linear decreasing approaches perform better than the fixed step method. Secondly, they proposed self-exploration behaviour for GSO. In this variant, they suggested that each glowworm is assigned with a threshold and the fitness value should be greater than this value for a glowworm and also its neighbours. If not, the glowworm needs to choose randomly between random spiral search and random Z-shaped search in order to find better fitness value. If the fitness value is greater than the threshold then the basic GSO algorithm is used [100]. Zhao *et al*. [101] introduced a local search operator to GSO with an aim to increase convergence accuracy and efficiency [101].

### Cuckoo Search Algorithm

The Cuckoo Search Algorithm (CSA) is one of the latest metaheuristic approaches introduced by Yang and Deb in 2009 [102]. This algorithm is inspired by the behaviour of cuckoo species, such as brood parasites, and the characteristics of Lévy flights, such as some birds and fruit flies. CSA employs three basic rules or operations in its implementation. First, each cuckoo is only allowed to lay one egg in each iteration, and the nest is chosen randomly by the cuckoo to lay its egg in. Second, the eggs and nests with high quality are carried forward to the next generation. Third, the number of available host nests is fixed and the egg laid by a cuckoo is discovered by a host bird using probability *p*_a_ϵ [0, 1]. In other words, the host can choose whether to throw the egg away or abandon the nest and build a new nest completely. The last assumption can be approximated as a fraction, *p*_*a*_ of the total *n* nests that are replaced by new nests with a new random solution. The algorithm also can be extended to more complicated point where each nest contains multiple eggs [102, 103]. Based on these three main rules the details of steps taken in CSA are discussed. To generate a new solution, *x* (*t*+1), for cuckoo indexed *m*, the following Levy flight equation is performed [102–104]:

$$x_{m}\left(t+1\right)=x_{m}\left(t\right)+\partial ⊕Levy\left(\beta \right)$$ (19)

where *∂* is the step size. In most cases, *∂* = 1 is used [102]. The product ⊕ is an indication of matrix form multiplication and using entry-wise approach. Levy flights provide a random walk and the random steps are drawn from a Levy Distribution equation for large steps as follows:

$$Levy\sim u=t^{-1-\beta }(0<\beta <2)$$ (20)

The equation has infinite variance with an infinite mean. The following steps of a cuckoo from a random walk process are required to fulfil step-length distribution with a heavy tail. A fraction, *p*_*a*_, of the worst nest is discarded therefore the new nests can be built at new locations. The mixing of the solutions is performed by random permutation depending on similarity or difference to the host eggs. The step size, *∂*, initializes with a large value and iteratively decreases towards the final generation allowing the population to be converged towards a solution in the final generation. In principles this is similar to the steps taken in linear decreasing PSO. The additional component is introduced to Eq (19) and form Eq (21) by Yang [104]:

xm(t+1)=xm(t)+∂⊕Levy(β)~0.01u|v|1β(xn(t)−xm(t)) (21)

where *u* and *v* are drawn from normal distribution which is

$$u\sim N\left(0,\sigma_{u}^{2}\right),\;v\sim N\left(0,\sigma_{u}^{2}\right)$$ (22)

where

$$\sigma_{u}={\left\{\frac{\left(\gamma \left(1+\beta \right)\sin\left(\frac{\pi \beta }{2}\right)\right)}{\left(\gamma \left[1+\beta \right)/2\right]\beta 2^{\frac{\beta -1}{2}}}\right\}}^{{}^{1/\beta }},\sigma_{v}=1$$ (23)

γ is the standard gamma function [104]. The exploration occurs if there is a large difference value between *x*_*n*_ and *x*_*m*_ in Eq 21 and a small difference results in an exploitation.

CSA is advantageous with multimodal objective functions and it requires fewer numbers of parameters to be fine-tuned compared to other approaches. It also has an insensitive convergence rate to the parameter *p*_a_ where on some occasions fine tuning the parameters is not necessary [102–104]. CSA is applied to various areas including neural network [105], embedded systems [106], electromagnetics [107], economics [108], business [109], and TSP problem [110].

In 2011, Walton et al. have introduced a variant for CSA called Modified Cuckoo Search (MCS) where their main objective is to increase the convergence speed [111]. This enhancement involves an additional step in which the top eggs do some information sharing. They have applied MCS on several benchmark functions and the results show that MCS has outperformed the standard CSA. The other popular variant for CSA is Quantum Inspired Cuckoo Search Algorithm (QICSA) proposed by Layeb in 2011 [112]. The author integrated elements from quantum computing principles like qubit representation, measure operation, and quantum mutation. The main objectives are to enhance the diversity and the performance of standard CSA. The results showed that there are still some shortcomings in QICSA and the author suggested to integrate a local search and parallel machines in order to improve the efficiency and increase the convergence speed [112].

### Other Evolutionary Algorithms

There are so many other evolutionary algorithms available but not discussed in the previous sections because the purpose of the previous section were to only introduce and discuss the well-known and commonly used SI-based approaches. Therefore, this section is dedicated to discuss in general the other interesting evolutionary algorithms such as Genetic Programming (GP), Evolution Strategy (ES), Evolutionary Programming (EP), Firefly Algorithm (FA), Bat Algorithm (BA) and Grey Wolf Optimizer (GWO).

GP is another evolutionary algorithm which involves similar procedures taken in GA. GP uses the term *program* while GA uses the term *chromosome* to represent the solution. The procedures for GP start with creating an initial population randomly. Later, three steps are repeated until the stopping criteria is met. These steps are *fitness evaluation*, *selection* and *reproduction*. The only difference between GP and GA is in selection procedure. GA selects predefined percentages of the fittest population for reproduction while in GP, each program selects one program or a few programs (according to the objective) from the population depending on the probability assigned to each program (based on their fitness) [113].

The ES algorithm is another type of optimization approach that uses the same methodology as GA and DE but it utilizes self-adaptive mutation rates. It has three types of procedures which are (1+1)-ES, (1+λ)-ES and (μ/ρ +, λ)-ES. (1+1)-ES operates where each parent produces just one mutation (child) who competes with that parent. The mutant will become the parent on the next generation only if it performs as well as the original parent. If not, then the mutant is omitted. In (1+λ)-ES, λ mutants are generated and the best mutant is selected as the new parent in the next generation while the current parent is omitted without considering its fitness. (μ/ρ +, λ)-ES is quite contemporary and often used as standard ES. μ represents number of individuals contained in the parent population and ρ it the decided numbers of parent individuals used for recombination. Hence, ρ should be equal or less than μ. λ represents the number of child produced in each generation. Note that all of these parameters are positive integers. +, is the operator to decide which strategy applies whether ‘plus’ or ‘comma’ strategy. ‘Plus’ strategy neglects the age of individuals, meaning that parents are competing with their children to survive and be bought to next generation. ‘Comma’ strategy is where the parents are always omitted and new parents are chosen from the fittest child for new generation [114].

EP shares the same similarities with the steps taken in GA which involves initialization, mutation and evaluation operations. However, the main difference between EP and GA is where EP does not use any crossover operation to generate child or offspring. EP and ES share a lot of similarities between them. However, they have two main differences which are in selection and recombination. EP usually uses stochastic selection and ES uses deterministic selection. Stochastic selection means that each solution competes against a predetermined number of other solutions and the least-fit solutions are eliminated. Deterministic selection means it eliminate the worst solutions directly after their evaluation [115, 116]. FA was inspired by the behaviour of fireflies which attract each other using flashing light. FA is quite similar to GSO algorithm in terms of inspiration. The fitness of the fireflies will determine their flashing brightness. This brightness also decreases over distance. The less bright firefly will move towards a firefly which is brighter, and if there is no brighter firefly, the particular firefly will move randomly [117].

The Bat algorithm is another recent introduced optimization technique. It is introduced by Yang and Gandomi in 2012 and it is inspired form bats behaviour in foraging for food. This algorithm is quite similar to PSO and it is consist of velocity and position equations [118, 119]. Since this algorithm is inspired by bats, it considers the echolocation capability that bats have and also take advantage of a frequency equation. This frequency equation has direct influence on the velocity equation which determines the direction in search space [118–120].

Mirjalili *et al*. introduced GWO which inspired by the predator grey wolf [121]. The algorithm divides the agents (grey wolves) into several categories of hierarchy named alpha, beta, delta and omega from top to bottom respectively. Each hierarchy has different roles in order to find the solutions, which in this case are preys [121]. Note that there are many more evolutionary algorithms that are not discuss in this paper. Mirjalili *et al*. listed some existing optimization algorithms that have not been discussed in this paper [121].

## Benchmark Functions Experiment

There are many optimization techniques claiming superiority over other approaches. Hence, to determine the most reliable algorithms, benchmark functions can be used as indicator to prove their effectiveness. Several benchmark functions with different properties have been used to evaluate the feasibility of the discussed optimization algorithms; their achieved performances are presented in this section. There are four experiments which have been done. The first experiment is the comparison between seven algorithms discussed with more rigorous conditions in order to determine the best basic evolutionary algorithm. The second and third experiments are the variants of the two best evolutionary algorithms based on the performance from the first experiment. The fourth experiment is available in supplement section where the comparison between seven algorithms discussed on twenty benchmark functions with all results being collected from literature [140–157]. The fifth experiment which is also available in the S1 File discusses the behaviour of all these algorithm when an offset is added into the function.

### Experimental Settings

In evolutionary methods, experimental settings are very important and can influence the outcome of the experiments. If the settings are not optimal then the outputs are not optimal either. In order to have a fair assessment between all algorithms, it is important to set the value of each algorithm to its optimal value. Optimal setting means that the best setting is used in order to obtain the best possible result. For example in the PSO algorithm, linear decreasing inertia weight should be used instead of random inertia weight to balance the exploration and exploitation behaviour of the particles which increase the chance of obtaining the global optima. There are several studies discussed about the optimal value. They have run a number of experiments to obtain the optimal setting value in order to get the best possible outcome from the optimization problems. For example, Fernando et. al [122] investigated parameter setting in genetic algorithm. Parapar, Vidal and Santos [123] discussed how to find the best parameter setting for PSO and Zhang, Yu and Hu [124] have suggested the optimal choice of fix inertia weight value for PSO. Josef [125] and Zhang et. al [126] have recommended the best parameter setting for DE and GSO respectively. For ABC, Akay and Karaboga [127] have suggested to tune the parameter for the best optimal result. Gaertner and Clrk [128] and Stutzle et. al [129] investigated ways to set the parameters for ACO. Various types of benchmark functions and settings are used for the evaluation. First, the settings of each comparison are discussed and later, the benchmark functions selected are presented.

#### Experiment 1: Performance Evaluation on Benchmark Functions with strict conditions

The performance of seven optimization algorithms (GA, ACO, DE, PSO, ABC, GSO, and CSA) discussed earlier are compared against each other with rigorous conditions applied. The aim of this experiment is to distinguish which optimization algorithm can give the best performance in terms of outcome and time taken with limited iterations. In order to have fair comparison of performance among the evolutionary methods, all methods have the same iteration numbers and population sizes which is one hundred. The experimental settings utilized in this study are presented in Table 1. The benchmark functions selected and their characteristics are presented in Table 2 [130, 131].

**Table 1:** Experimental settings of the utilized methods.

Method	Settings Details
GA	Single point crossover type is used with 1 crossover probability. Mutation probability is set to 0.01 and 2 best solutions are selected for elitism.
ACO	Initial pheromone value used is 1.0E-06 with *Q* (pheromone update constant) is 20 and *q*_*0*_ (exploration constant) is 1. Global pheromone decay rate is 0.9 and local pheromone decay rate is 0.5. α used is 0.5 and β used is 2.5.
PSO	Inertia weight value used is 0.728994 with acceleration coefficients for cognitive and social are 0.5 and 2.5 respectively.
DE	Crossover constant used is 0.9 with type of DE used is DE/*best*/1/*bin*.
ABC	The number of colony size used is 100. The number of food sources is half of the colony size. The limit value is 10 where after that, the food source will be abandoned by employed bee if there is no improvement at all.
GSO	Number of max neighbour considered, *N* is set to 12 with γ value is 0.64. The ρ value is 0.35 and the β value is 0.45
CSA	Number of nests used is 25 with discover rate of alien eggs, *p_a_* is 0.25. Tolerance value is set to 1.0E-05.

**Table 2:** Benchmark Functions Selected for Comparison.

No	Function	Formula	Value	Dim	Range	Properties
1	Sumsquare	$f\left(x\right)=\sum_{i=1}^{n}ix_{i}^{2}$	0	30	[-5.12, 5.12]	Unimodal, Separable
2	Sphere	$f\left(x\right)=\sum_{i=1}^{n}x_{i}^{2}$	0	30	[–100, 100]	Unimodal, Separable
3	Beale	$f\left(x\right)={\left(1.5-x_{1}+x_{1}x_{2}\right)}^{2}+{\left(2.25-x_{1}+x_{1}x_{2}^{2}\right)}^{2}+{\left(2.625-x_{1}+x_{1}x_{2}^{3}\right)}^{2}$	0	2	[-4.5, 4.5]	Unimodal, Inseparable
4	Colville	$\begin{array}{l} f\left(x\right)=100{\left(x_{1}^{2}-x_{2}\right)}^{2}+{\left(x_{1}-1\right)}^{2}\\ +{\left(x_{3}-1\right)}^{2}+90{\left(x_{3}^{2}-x_{4}\right)}^{2}\\ +10.1({\left(x_{2}-1\right)}^{2}+{\left(x_{4}-1\right)}^{2})\\ +19.8\left(x_{2}-1\right)\left(x_{4}-1\right) \end{array}$	0	4	[–10, –10]	Unimodal, Inseparable
5	Dixon-Price	$f\left(x\right)=-{\left(x_{1}-1\right)}^{2}+\sum_{i=0}^{n}i{\left(2x_{i}^{2}-x_{i}-1\right)}^{2}$	0	24	[–5, 5]	Unimodal, Inseparable
6	Easom	$\begin{array}{l} f\left(x\right)=-cos\left(x_{1}\right)cos\left(x_{2}\right)\\ \text{exp}\left(-{\left(x_{1}-\pi \right)}^{2}-{\left(x_{2}-\pi \right)}^{2}\right) \end{array}$	0	30	[–30, 30]	Unimodal, Inseparable
7	Matyas	$f\left(x\right)=0.26(x_{1}^{2}+x_{2}^{2})-0.48x_{1}x_{2}$	0	2	[–10, 10]	Unimodal, Inseparable
8	Powell	$\begin{array}{l} f(x)=\sum {}_{\left(i=1\right)}^{\left(n/k\right)}{\left(x_{\left(4i-3\right)}+10x_{\left(4i-2\right)}\right)}^{2}\\ +5{\left(x_{\left(4i-1\right)}+x_{4}i\right)}^{2}+{\left(x_{\left(4i-2\right)}+x_{\left(4i-1\right)}\right)}^{4}\\ +10{\left(x_{\left(4i-3\right)}+x_{4}i\right)}^{4} \end{array}$	0	2	[–100, 100]	Unimodal, Inseparable
9	Rosenbrock	$f\left(x\right)=\sum_{i=1}^{n-1}[100{\left(x_{i+1}-x_{i}^{2}\right)}^{2}+{\left(x_{i}-1\right)}^{2}]$	-1	2	[–100, 100]	Unimodal, Inseparable
10	Schwefel	$f\left(x\right)=\sum_{i=1}^{n}-x_{i}\sin\left(\sqrt{\left|x_{i}\right|}\right)$	0	30	[–500, 500]	Unimodal, Inseparable
11	Trid 6	$f\left(x\right)=\sum_{i=1}^{n}{\left(x_{i}-1\right)}^{2}-\sum_{i=1}^{n}x_{i}x_{i-1}$	-50	6	[-D^2^, D^2^]	Unimodal, Inseparable
12	Zakharov	$f\left(x\right)=\sum_{i=1}^{n}x_{i}+{\left(\sum_{i=1}^{n}0.5ix_{i}\right)}^{2}+{\left(\sum_{i=1}^{n}0.5ix_{i}\right)}^{4}$	0	10	[–5, 10]	Unimodal, Inseparable
13	Bohachevsky1	$f\left(x\right)=x_{1}^{2}+2x_{2}^{2}-0.3cos\left(3\pi x_{1}\right)-0.4cos\left(4\pi x_{2}\right)+0.7$	0	2	[–100, 100]	Multimodal, Separable
14	Booth	*f*(*x*) = (*x*_1_ + 2*x*_2_ − 7)^2^ + (2*x*_1_ + *x*_2_ − 5)^2^	0	2	[–10, 10]	Multimodal, Separable
15	Branin	$f\left(x\right)={\left(x_{2}-\frac{5.1}{4\pi^{2}}x_{1}^{2}+\frac{5}{\pi }x_{1}^{2}-6\right)}^{2}+10(1-\frac{1}{8\pi })\cos x_{1}+10$	0.398	2	[–5, 10] x [0, 15]	Multimodal, Separable
16	Michalewicz5	$f\left(x\right)=-\sum_{i=1}^{n}\sin\left(x_{i}\right){\left(\sin\left(ix_{i}^{2}/\pi \right)\right)}^{2m}$	-4.688	5	[0, π]	Multimodal, Separable
17	Rastrigin	$f\left(x\right)=\sum_{i=1}^{n}x_{i}^{2}-10\cos\left(2\pi x_{i}\right)+10$	0	30	[-5.12, 5.12]	Multimodal, Separable
18	Shubert	$f\left(x\right)=\left(\sum_{i=1}^{5}i\cos\left((i+1)x_{1}+i\right)\right)\left(\sum_{i=1}^{5}i\cos\left((i+1)x_{2}+i\right)\right)$	-186.73	2	[–10, 10]	Multimodal, Separable
19	Ackley	$f\left(x\right)=-20\exp\left(-0.2\sqrt{\frac{1}{n}\sum_{i=1}^{n}x_{i}^{2}}\right)-\exp\left(\frac{1}{n}\sum_{i=1}^{n}\cos(2\pi x_{i})\right)+20+e$	0	30	[–32, 32]	Multimodal, Inseparable
20	Bohachevsky2	$f\left(x\right)=x_{1}^{2}+2x_{2}^{2}-0.3cos\left(3\pi x_{1}\right)cos\left(4\pi x_{2}\right)+0.3$	0	2	[–100, 100]	Multimodal, Inseparable
21	Bohachevsky3	$f\left(x\right)=x_{1}^{2}+2x_{2}^{2}-0.3cos\left(3\pi x_{1}+4\pi x_{2}\right)+0.3$	0	2	[–100, 100]	Multimodal, Inseparable
22	Bukin 6	$f\left(x\right)=100\sqrt{\left|x_{2}-0.01x_{1}^{2}\right|}+0.01|x_{1}+10|$	0	2	*x*_*1*_ ∈ [–15, –5], *x*_*2*_ ∈ [–3, 3]	Multimodal, Inseparable
23	Drop-Wave	$f\left(x\right)=-\frac{1+\cos\left(12\sqrt{x_{1}^{2}+x_{2}^{2}}\right)}{0.5(x_{1}^{2}+x_{2}^{2})+2}$	-1	2	[-5.12, 5.12]	Multimodal, Inseparable
24	Eggholder	$f\left(x\right)=-(x_{2}+47)\sin\left(\sqrt{\left|x_{2}+\frac{x_{1}}{2}+47\right|}\right)-x_{1}\sin\left(\sqrt{\left|x_{1}+(x_{2}+47\right|}\right)$	-959.6407	2	[-5.12, 5.12]	Multimodal, Inseparable
25	GoldStein-Price	$f\left(x\right)=\left[1+{\left(x_{1}+x_{2}+1\right)}^{2}(19-14x_{1}+3x_{1}^{2}-14x_{2}+6x_{1}x_{2}+3x_{2}^{2}\right]$ $\times \left[30+{\left(2x_{1}-3x_{2}\right)}^{2}(18-32x_{1}+12x_{1}^{2}+48x_{2}-36x_{1}x_{2}+27x_{2}^{2}\right]$	0	2	[–10, 10]	Multimodal, Inseparable
26	Griewank	$f\left(x\right)=\frac{1}{4000}\sum_{i=1}^{n}x_{i}^{2}-\prod_{i=1}^{n}\cos\frac{x_{i}}{\sqrt{i}}+1$	0	30	[–600, 600]	Multimodal, Inseparable
27	McCormick	*f*(*x*) = sin(*x*_1_ + *x*_2_) + (*x*_1_ + *x*_2_)^2^ − 1.5*x*_1_ + *x*2.5_2_ + 1	-1.9133	2	*x*_*1*_ ∈ [-1.5, 4], *x*_*2*_ ∈ [–3, 4]	Multimodal, Inseparable
28	Perm	$f\left(x\right)=\sum_{k=1}^{n}\sum_{i=1}^{n}{\left(i^{k}+\beta ){\left(x_{i}/i\right)}^{k}-1\right)}^{2}$	0	4	[-D, D]	Multimodal, Inseparable
29	Schaffer 2	$f\left(x\right)=0.5+\frac{\sin^{2}(\sqrt{x_{1}^{2}+x_{2}^{2}})-0.5}{{\left(1+0.0001\left(\sqrt{x_{1}^{2}+x_{2}^{2}}\right)\right)}^{2}}$	0	2	[–100, 100]	Multimodal, Inseparable
30	Schaffer 4	$f\left(x\right)=0.5+\frac{\cos(\sin(|x_{1}^{2}+x_{2}^{2}))-0.5}{{\left(1+0.0001\left(x_{1}^{2}+x_{2}^{2}\right)\right)}^{2}}$	0	2	[–100, 100]	Multimodal, Inseparable

#### Experiment 2: Performance Evaluation on Benchmark Functions between several modified DE algorithms

Various modified DE-based algorithms are considered to assess their performance against the basic DE approach. The selected DE-based algorithms are Strategy Adaption Differential Evolution (SADE) [132], Adaptive Differential Evolution with Optional External Archive (JADE) [133], Opposition-based Differential Evolution (OBDE) [134], and Compact Differential Evolution (cDE) [135] together with basic Differential Evolution (DE). The performance evaluation is assessed based on the reported fitness on six benchmark functions including Sphere, Rosenbrock, Schwefek, Rastrigin, Michalewicz5 and Griewank functions.

#### Experiment 3: Performance Evaluation on Benchmark Functions between several modified PSO algorithms

Four modified PSO algorithms are considered to assess their performance against the basic PSO method. The selected approaches include Selection PSO (SPSO) [136], Compact PSO (cPSO) [137], Intelligence Single PSO (ISPSO) [138], and Comprehensive Learning PSO (CLPSO) [139] along with original PSO. The performances of the chosen approaches are assessed based on their fitness on a similar set of benchmark functions to those that have been used in experiment 2 (Sphere, Rosenbrock, Schwefek, Rastrigin, Michalewicz5 and Griewank functions).

### Benchmark Functions

Every benchmark function has its own properties whether it is unimodal, multimodal, separable or non-separable. It is noteworthy that the combination of these properties determines the complexity of the functions. A function is considered multimodal if it has two or more local optima and it is considered separable if it can be rewritten as a sum of function just from one variable. The concept of epistasis or interrelation between variables of the function is related to separable properties. The concept of epistasis is a concept of genetics where the outcome of one genetic factor can be governed by the existence of one or more modified genetic factor. The problem becomes more complicated if the function is multimodal as well. The global optimum is the value that needs to be estimated during the search process, therefore, the regions around local minima must be avoided as far as possible. If the local optima are distributed randomly in the search area, it is considered as the most difficult problem. The aim of optimization process is to obtain the global optima, therefore the regions around local optima should be avoided because the swarm might get stuck in local optima and consider that local optima as the global optima. Another important property that determines the difficulty of the problem is the dimension of the search area. Table 2 presents the list of benchmark functions utilized to assess the performance of the considered evolutionary methods. The table consists of the name of the benchmark function, the range, the dimension, the characteristic of the function and its formula. The characteristic of the function determines the complexity of the function.

## Comparisons and Discussion

The reported results in this section do not necessarily reflect the performance of the utilized methods under all circumstances. The overall performance of such methods can be influenced by the utilized parameterizations and other experimental conditions. However, benchmark functions can be the indicators of how well the optimization algorithms perform under several degrees of complexities. In this section, the results of all selected algorithms tested on thirty benchmark functions are presented and discussed.

### Performance Evaluation on Benchmark Functions

In this experiment, the performance of optimization techniques selected are assessed on a variety of benchmark functions using MATLAB2011 on a CORE i5 CPU with 2GB RAM and have been run thirty times. The average result of the runs (Mean), standard deviation (SD) and time taken (in seconds) to complete each run are reported in Tables 3, 4 and 5. If the mean value is less than 1.000E-10, then the result is reported as 0.000E+00. In this experiment, only basic versions of SI techniques are considered and no modifications are applied. Algorithm codes are adapted from several sources and are modified to be compatible with our experimental setup [158–161].

**Table 3:** Benchmark Functions Comparison of mean error (Mean ± SD) and time (Seconds) on Several Optimization Techniques.

Function	GA	ACO	PSO	DE
Sphere (Separable)	6.4415E+03	1.7596E+04	1.0454E+05	**5.5942E+03**
	±1.6876E+03	±1.8603E+03	±7.1998E+04	**±1.5091E+03**
	(4.3531s)	(7.3219s)	(2.8906s)	(10.9984s)
Sumsquare (Separable)	1.7376E+01	5.6363E+00	**3.7357E+00**	7.7637E+00
	±3.7449E-15	±4.0719E-01	**±1.8203E-01**	±1.4868E+00
	(3.8938s)	(6.9031s)	**(3.1422s)**	(11.4047s)
Beale (Inseparable)	7.0313E-01	7.0313E-01	**0.0000E+00**	**0.0000E+00**
	±0.0000E+00	±0.0000E+00	**±0.0000E+00**	**±0.0000E+00**
	(3.1078s)	(2.8938s)	(1.9094s)	(4.9531s)
Colville (Inseparable)	**0.0000E+00**	6.6160E+01	**0.0000E+00**	1.4017E+00
	**±0.0000E+00**	±3.7940E+01	**±0.0000E+00**	±2.1101E+00
	(2.6875s)	(2.2703s)	(1.8922s)	(4.7859s)
Dixon-Price (Inseparable)	1.1029E+05	1.8708E+06	4.9633E+06	**1.3145E+04**
	±3.4184E+04	±4.3444E+05	±3.4317E+06	**±6.3822E+03**
	(4.1063s)	(6.7469s)	(3.1000s)	(11.3109s)
Easom (Inseparable)	**-1.0000E+00**	**-1.0000E+00**	**-1.0000E+00**	**-1.0000E+00**
	**±0.0000E+00**	**±0.0000E+00**	**±0.0000E+00**	**±0.0000E+00**
	(6.0281s)	(2.0016s)	(1.8875s)	(4.8734s)
Matyas (Inseparable)	**0.0000E+00**	**0.0000E+00**	**0.0000E+00**	**0.0000E+00**
	**±0.0000E+00**	**±0.0000E+00**	**±0.0000E+00**	**±0.0000E+00**
	(2.7453s)	(2.0500s)	(2.0250s)	(5.0875s)
Powell (Inseparable)	4.2230E+02	9.4665E+03	1.0689E+04	**3.1662E+02**
	±1.2382E+02	±1.4600E+03	±3.7167E+03	**±1.3003E+02**
	(3.9266s)	(5.5594s)	(2.6859s)	(9.4516s)
Rosenbrock (Inseparable)	1.2493E+07	1.1051E+08	7.0289E+08	**3.8901E+06**
	±8.6725E+06	±2.1694E+07	±4.8937E+08	**±2.2417E+06**
	(4.0797s)	(6.9359s)	(2.9797s)	(11.0344s)
Schwefel (Inseparable)	5.2808E+03	**3.2250E+03**	7.0202E+03	5.6371E+03
	±6.2830E+02	**±4.5211E+02**	±1.2171E+02	±5.9306E+02
	(4.4391s)	(9.9703s)	(2.8094s)	(12.5828s)
Trid6 (Inseparable)	-2.5000E+01	-2.4300E+01	**-5.0000E+01**	-4.7697E+01
	±1.2293E+01	±9.3339E+00	**±0.0000E+00**	±5.0327E+00
	(3.1516s)	(2.7422s)	(2.0859s)	(5.8891s)
Zakharov (Inseparable)	2.9550E+01	7.1088E+01	**0.0000E+00**	**0.0000E+00**
	±2.0370E+01	±1.4866E+01	**±0.0000E+00**	**±0.0000E+00**
	(3.8078s)	(3.4719s)	(2.3953s)	(6.8844s)

**Table 4:** Benchmark Functions Comparison of mean error (Mean ± SD) and time (Seconds) on Several Optimization Techniques.

Function	ABC	GSO	CSA	p-value
Sphere (Separable)	1.1820E+05	1.1844E+06	4.4138E+04	0.0001
	±8.3508E+03	±8.0723E+04	±5.5047E+04	
	(0.5641s)	(12.3234s)	(2.0959s)	
Sumsquare (Separable)	1.7476E+01	2.0526E+01	1.6531E+01	0.0001
	±3.1623E-01	±2.9771E-01	±7.9493E-01	
	(0.7153s)	(12.1047s)	(2.1822s)	
Beale (Inseparable)	**0.0000E+00**	1.7223E+00	6.5750E-02	0.0001
	**±0.0000E+00**	±6.0540E-02	±2.1365E-02	
	(0.7496s)	(4.9531s)	(1.5618s)	
Colville (Inseparable)	7.3760E+01	1.1701E+02	6.4181E+01	0.0001
	±2.8049E+01	±2.6130E+01	±5.5250E+00	
	(0.4483s)	(6.7732s)	(1.4921s)	
Dixon-Price (Inseparable)	2.2939E+06	3.1354E+08	8.1887E+05	0.0145
	±1.5742E+06	±3.6645E+08	±2.4639E+06	
	(6.7469s)	(14.2784s)	(2.2086s)	
Easom (Inseparable)	1.8974E-03	1.0670E+00	-1.7374E-03	0.001
	±1.2470E-04	±5.3736E-02	±5.6839E-04	
	(0.5687s)	(6.4734s)	(1.2498s)	
Matyas (Inseparable)	**0.0000E+00**	2.4540E+00	**0.0000E+00**	0.001
	**±0.0000E+00**	±2.6413E-01	**±0.0000E+00**	
	(1.8700s)	(7.0736s)	(1.9967s)	
Powell (Inseparable)	2.6977E+05	3.6742E+06	1.0722E+04	0.0913
	±4.3360E+05	±6.5117E+06	±3.7790E+03	
	(0.5690s)	(11.5516s)	(1.7003s)	
Rosenbrock (Inseparable)	4.8807E+10	1.7626E+12	1.2493E+07	0.0592
	±1.1684E+10	±2.7674E+12	±8.6725E+06	
	(0.6313s)	(15.2344s)	(2.0009s)	
Schwefel (Inseparable)	3.6619E+03	7.7821E+04	6.6619E+03	0.001
	±2.3244E+02	±2.0826E+03	±4.1047E+02	
	(0.9188s)	(14.6285s)	(1.7777s)	
Trid6 (Inseparable)	-2.6800E+01	-1.5100E+01	-3.3100E+01	0.001
	±2.8206E+00	±1.1836E+01	±3.2813E+00	
	(0.5126s)	(7.1442s)	(2.7422s)	
Zakharov (Inseparable)	9.2688E+01	1.3259E+02	1.8454E+01	0.001
	±5.5187E+00	±1.1658E+01	±2.9051E+00	
	(0.6348s)	(8.7891s)	(1.3908s)	

**Table 5:** Benchmark Functions Comparison of mean error (Mean ± SD) and time (Seconds) on Several Optimization Techniques.

Function	GA	ACO	PSO	DE
Bohachecvsky1 (Separable)	**0.0000E+00**	**0.0000E+00**	**0.0000E+00**	**0.0000E+00**
	**±0.0000E+00**	**±0.0000E+00**	**±0.0000E+00**	**±0.0000E+00**
	(6.3516s)	(1.8641s)	(1.9328s)	(5.1844s)
Booth (Separable)	**0.0000E+00**	**0.0000E+00**	**0.0000E+00**	**0.0000E+00**
	**±0.0000E+00**	**±0.0000E+00**	**±0.0000E+00**	**±0.0000E+00**
	(2.5125s)	(1.8719s)	(1.8234s)	(4.7984s)
Branin (Separable)	**3.9789E-01**	**3.9789E-01**	**3.9789E-01**	**3.9789E-01**
	**±0.0000E+00**	**±0.0000E+00**	**±0.0000E+00**	**±0.0000E+00**
	(5.9844s)	(1.8563s)	(1.9719s)	(4.9469s)
Michalewciz5 (Separable)	-1.5651E+00	-1.5651E+00	-1.1906E+00	**-4.1803E+00**
	±0.0000E+00	±0.0000E+00	±3.3261E-01	**±4.2335E-01**
	(2.6859s)	(2.5172s)	(1.9797s)	(5.3094s)
Rastrigin (Separable)	**5.5900E+01**	1.7840E+02	5.4130E+02	1.8730E+02
	**±1.4294E+01**	±2.5299E+01	±1.5969E+01	±1.9989E+01
	(3.7891s)	(6.8125s)	(2.9531s)	(10.1328s)
Shubert (Separable)	-1.2884E+02	-1.2884E+02	-**1.8673E+02**	-**1.8673E+02**
	±0.0000E+00	±0.0000E+00	**±0.0000E+00**	**±0.0000E+00**
	(3.2000s)	(1.8266s)	(1.9844s)	(4.9500s)
Ackley (Inseparable)	1.7194E+01	1.5884E+01	1.6004E+01	**1.2795E+01**
	±7.9083E-01	±1.2211E+00	±5.3105E+00	**±8.4147E-01**
	(4.0344s)	(8.9734s)	(2.9844s)	(11.5375s)
Bohachecvsky2 (Inseparable)	**0.0000E+00**	**0.0000E+00**	**0.0000E+00**	**0.0000E+00**
	**±0.0000E+00**	**±0.0000E+00**	**±0.0000E+00**	**±0.0000E+00**
	(5.7203s)	(1.8516s)	(2.0359s)	(5.0547s)
Bohachecvsky3 (Inseparable)	**0.0000E+00**	**0.0000E+00**	**0.0000E+00**	**0.0000E+00**
	**±0.0000E+00**	**±0.0000E+00**	**±0.0000E+00**	**±0.0000E+00**
	(5.5688s)	(1.8547s)	(1.9438s)	(5.0203s)

#### Tables 3 and 4

The first two benchmark functions in Table 3 and Table 4 (e.g., Sphere and Sumsquare) are unimodal and separable with a theoretical minimization value of zero. In Sphere, the result which is closest to the theoretical optimal value is acquired by DE with 5.5942E+03 and GA becomes the second best with 6.4415E+03. In the Sumsquare function, none of the algorithms achieved the best minimization performance but PSO has become the best algorithm with 3.7357E+00 and ACO has become the second best with 5.6363E+00. The third best is DE where it managed to achieve 7.7637E+00. The next ten functions in these tables are unimodal and inseparable (Beale, Coville, Dixon-Price, Easom, Matyas, Powell, Rosenbrock, Schwefel, Trid6 and Zakharov). The results in Beale function indicated that PSO, DE and ABC have achieved the optimal value (0.0) followed by CSA with 6.5750E-02. PSO and GA achieve better minimization performance compared with the other approaches when applied to the Coville functions (with zero being the optimal value). DE is the best performing method on the Dixon-Price function with the mean value of 1.3145E+04 followed by GA with the mean value of 1.1029E+05. In Easom function, GA, ACO, PSO and DE are the best performing approaches with all of them recording -1.0000E+00 mean value. All algorithms managed to achieve the theoretical optimal value with the Matyas function except for GSO where the mean value obtained is 2.4540E+00. DE managed to outperform other approaches by achieving 3.1662E+02 mean value which is the closest to the theoretical optimum value of -1 on the Powell function. DE and ACO are the best performing approaches on Rosenbrock and Schwefel functions respectively with the mean value of 3.8901E+06 and 3.2250E+03. In Trid6 function the theoretical value of -50, PSO manage to achieved this theoretical value and outperform the other algorithms. The second best algorithm is DE with the mean value of -4.7697E+01. In Zakharov function, PSO and DE managed to perform best by obtaining the theoretical value of 0. Considering the reported results in Table 3, DE is the best performing since it managed to be selected as the best approach with eight out of twelve functions closely followed by PSO, being selected as the best approach in seven out of twelve function. The third best approach for unimodal functions are GA and ACO where both of them have been selected as the best approach for three out of twelve functions. From all these considered methods, GSO is the poorest performing method due to not being able to become the best performing method in any of the functions. This is closely followed by CSA being selected as the best performing method in only one function. However, from literature investigation, ABC and CSA perform quite well when the number of evolutions were higher [140, 141]. They even managed to outperform other algorithms in several benchmark functions. Further discussion is available in the S1 File.

#### Tables 5, 6, 7 and 8

Tables 5 to 8 are focusing on multimodal functions with the first six functions (Bohachecvsky1, Booth, Branin, Michalewicz5, Rastrigin and Shubert) being separable. The best performance for Bohachecvsky1 is shared between five algorithms of GA, ACO, PSO, DE and ABC where they managed to find the theoretical value of 0. The second and third functions utilized are Booth and Branin functions where in these functions the best performance is shared between six algorithms (GA, ACO, PSO, DE, ABC and CSA). The forth function is Michalewicz5 with theoretical value of -4.687658. DE achieved the closest average per value to the theoretical value with -4.1803. In Rastrigin with 0 theoretical value, GA managed to outperform other algorithms with the mean value of 5.5900E+01. In Shuber function, PSO and DE managed to acquire the theoretical value of -186.7309.

**Table 6:** Benchmark Functions Comparison of mean error (Mean ± SD) and time (Seconds) on Several Optimization Techniques.

Function	ABC	GSO	CSA	p-value
Bohachecvsky1 (Separable)	**0.0000E+00**	1.7640E+00	8.2066E-03	0.001
	**±0.0000E+00**	±8.0414E-02	±8.0204E-03	
	(0.5953s)	(6.9719s)	(1.0634s)	
Booth (Separable)	**0.0000E+00**	4.6000E+00	**0.0000E+00**	0.001
	**±0.0000E+00**	±2.3002E-01	**±0.0000E+00**	
	(0.5858s)	(4.7984s)	(1.0859s)	
Branin (Separable)	**3.9789E-01**	3.7481E+01	**3.9789E-01**	0.001
	**±0.0000E+00**	±8.6588E-01	**±0.0000E+00**	
	(0.4856s)	(6.4852s)	(1.0778s)	
Michalewciz5 (Separable)	-3.5684E+00	-9.9061E-01	-1.5436E+00	0.001
	±3.2433E-02	±2.5724E-01	±6.7793E-02	
	(0.5264s)	(6.1347s)	(1.9797s)	
Rastrigin (Separable)	1.2382E+05	1.2679E+08	1.3202E+05	0.001
	±1.1630E+04	±1.3932E+07	±1.6245E+04	
	(0.6391s)	(12.3106s)	(2.0863s)	
Shubert (Separable)	-1.2942E+01	-8.8424E+00	-2.7642E+01	0.001
	±3.1623E-01	±0.0000E+00	±2.1499E+00	
	(0.4758s)	(6.5500s)	(1.0811s)	
Ackley (Inseparable)	2.0681E+01	1.9896E+01	1.2795E+01	0.001
	±3.8721E-02	±5.3227E-01	±8.4147E-01	
	(0.9875s)	(12.1059s)	(0.9875s)	
Bohachecvsky2 (Inseparable)	4.7124E-01	3.0422E+01	5.4223E+00	0.001
	±2.8573E-01	±6.9014E+00	±2.6812E+00	
	(0.4566s)	(6.7005s)	(1.1120s)	
Bohachecvsky3 (Inseparable)	5.2233E-01	1.2818E+01	2.8223E+00	0.001
	±3.3498E-01	±4.6593E-01	± 4.6749E-01	
	(0.4595s)	(6.1463s)	(1.0485s)	

**Table 7:** Benchmark Functions Comparison of mean error (Mean ± SD) and time (Seconds) on Several Optimization Techniques.

Function	GA	ACO	PSO	DE
Bukin 6 (Inseparable)	**0.0000E+00**	**0.0000E+00**	**0.0000E+00**	**0.0000E+00**
	**±0.0000E+00**	**±0.0000E+00**	**±0.0000E+00**	**±0.0000E+00**
	(2.4766s)	(1.8250s)	(1.9625s)	(4.9813s)
Drop-Wave (Inseparable)	**-1.0000E+00**	**-1.0000E+00**	**-1.0000E+00**	**-1.0000E+00**
	**±0.0000E+00**	**±0.0000E+00**	**±0.0000E+00**	**±0.0000E+00**
	(2.7391s)	(2.3813s)	(1.9641s)	(5.3031s)
Egg Holder (Insepearable)	**-9.1540E+02**	-8.4202E+02	-8.9632E+02	-9.0219E+02
	**±3.0628E+01**	±5.5959E+01	±5.7481E+01	±6.0614E+01
	(2.8531s)	(2.6031s)	(1.9313s)	(5.2063s)
Goldstein-Price (Inseparable)	**3.0000E+00**	**3.0000E+00**	**3.0000E+00**	**3.0000E+00**
	**±0.0000E+00**	**±0.0000E+00**	**±0.0000E+00**	**±0.0000E+00**
	(2.4531s)	(2.6172s)	(1.8375s)	(5.0313s)
Griewank (Inseparable)	1.2194E+00	1.1711E+00	3.2000E+00	**1.1282E+00**
	±8.9937E-02	±2.9271E-02	±1.5451E+00	**±4.0468E-02**
	(4.0516s)	(10.9766s)	(3.0766s)	(11.8531s)
McCormick (Inseparable)	**-1.9134E+00**	**-1.9134E+00**	**-1.9133E+00**	**-1.9132E+00**
	**±0.0000E+00**	**±0.0000E+00**	**±0.0000E+00**	**±0.0000E+00**
	(2.7969s)	(2.3266s)	(1.8844s)	(5.0672s)
Perm (Inseparable)	7.2815E+02	7.2815E+02	2.9684E+04	**0.0000E+00**
	±0.0000E+00	±0.0000E+00	±1.8921E+04	**±0.0000E+00**
	(3.0047s)	(2.5734s)	(2.1234s)	(5.2578s)
Schaffer 2 (Inseparable)	3.9880E-04	1.4299E-02	**0.0000E+00**	**0.0000E+00**
	±8.4075E-04	±1.9474E-02	**±0.0000E+00**	**±0.0000E+00**
	(2.4000s)	(2.2672s)	**(2.0328s)**	**(5.3063s)**
Schaffer 4 (Inseparable)	**0.0000E+00**	**0.0000E+00**	**0.0000E+00**	**0.0000E+00**
	**±0.0000E+00**	**±0.0000E+00**	**±0.0000E+00**	**±0.0000E+00**
	(2.7844s)	(2.4375s)	(1.9438s)	(5.3531s)

**Table 8:** Benchmark Functions Comparison of mean error (Mean ± SD) and time (Seconds) on Several Optimization Techniques.

**Function**	**ABC**	**GSO**	**CSA**	**p-value**
Bukin 6 (Inseparable)	**0.0000E+00**	3.5842E+00	5.5644E-04	0.001
	**±0.0000E+00**	±1.0744E-01	±1.7146E-05	
	(0.5403s)	(7.3113s)	(1.0375s)	
Drop-Wave (Inseparable)	-2.6485E-01	3.2720E+00	-5.5375E-01	0.001
	±1.7913E-02	±2.6682E-02	±2.4066E-02	
	(0.7640s)	(8.6189s)	(1.3165s)	
Egg Holder (Insepearable)	-8.0087E+02	-4.0822E+01	-8.1346E+02	0.001
	±4.8686E+01	±6.5870E+00	±5.2962E+01	
	(0.9230s)	(7.4538s)	(1.4645s)	
Goldstein-Price (Inseparable)	**3.0000E+00**	6.7935E+00	**3.0000E+00**	0.001
	**±0.0000E+00**	±2.3954E-01	**±0.0000E+00**	
	(0.6580s)	(7.4520s)	(1.2443s)	
Griewank (Inseparable)	3.0996E+01	9.3869E+01	9.2549E+00	0.001
	±2.2269E+00	±3.0447E+00	±3.3997E-01	
	(1.1484s)	(13.9829s)	(2.1890s)	
McCormick (Inseparable)	-1.8428E+00	1.2761E+00	-1.8450E+00	0.001
	±2.3137E-02	±1.1802E-01	±2.0994E-02	
	(0.5780s)	(6.3157s)	(1.2875s)	
Perm (Inseparable)	6.6668E+05	3.5024E+06	2.9684E+04	0.001
	±2.0984E+05	±2.5331E+06	±1.8921E+04	
	(0.7153s)	(7.2672s)	(1.5224s)	
Schaffer 2 (Inseparable)	1.4111E-02	1.7486E+01	1.4498E-02	0.001
	±1.9614E-02	±8.5832E-01	±1.9321E-02	
	(0.5408s)	(7.2107s)	(1.6002s)	
Schaffer 4 (Inseparable)	1.5864E-02	1.7718E+01	8.2066E-03	0.001
	±1.8986E-02	±8.4297E-01	±8.0204E-03	
	(0.5408s)	(7.2107s)	(1.6309s)	

The rest of the functions considered in these tables are multimodal and inseparable. The functions that are this type of characteristic are Ackley, Bohachecvsky2, Bohachecvsky3, Bukin6, Drop-Wave, Egg Holder, Goldstein-Price, Griewank, McCormick, Perm, Schaffer2 and Schaffer4. Considering these functions, DE, became the best performing approach achieving the best performance in 11 out of 12 functions. DE has performed best in all functions except Egg-Holder where GA is the best performing approaches. In Egg-Holder, GA managed to record a mean value of -9.1540E+02 which is the closest to the optimal value of -959.6407. PSO and GA shared the second best performing approaches where they become the best algorithm in eight out of twelve function. It is noticeable that GA, ACO, PSO and DE share the best performing approaches in Bohachecvsky2, Bohachecvsky3, Bukin6, Drop-Wave, Goldstein-Price, McCormick and Schaffer functions where all of them managed to find the theoretical optimal value of zero. Within the Griewank and Perm functions, DE has become the best performing approach with a mean value of 1.1282E+00 and 0 respectively. PSO and DE once again have become the best methods when applied to Schaffer2 function where they managed to obtain an optimal value of 0.

#### Overall performance

The results presented in Tables 3 to 8 can also be investigated based on the characteristics of the fitness functions utilized in the study. Considering categories of i) Unimodal and Separable (US), ii) Unimodal and Inseparable (UI), iii) Multimodal and Separable (MS), iv) Multimodal and Inseparable (MI), v) Multimodal (M), vi) Unimodal (U), vii) Separable (S), and viii) Inseparable (I), Table 9 is formed. Considering the results presented in Table 9, DE seems to be the best overall performing approach, outperforming other methods in 24 out of 30 functions followed by PSO with the best performance in 19 out of 30. The third best is GA with 14 out 30 best performance and closely followed by ACO with 13 out of 30 best performance. ABC, and CSA reached the best performance in 6 and 3 out of 30 functions respectively. Focusing on the breakdown results it is noticeable that DE has been the best performing method in all categories. However, in terms of time consumed to complete the benchmark test, ABC is the best with an average for all 30 functions is 0.8850 seconds and followed by CSA with an average of 1.5738. Even DE is the best overall performance in term of mean value but it is the second slowest algorithm after GSO.

**Table 9:** Performance breakdown based on the benchmark functions’ characteristics.

Category	Number of functions	GA	ACO	PSO	DE	ABC	GSO	CSA
**Being best performing method**	30	15	13	19	24	6	0	3
**Unimodal Separable (US)**	2	0	0	1	1	0	0	0
**Unimodal Inseparable (UI)**	10	3	3	6	7	2	0	1
**Multimodal Separable (MS)**	6	4	3	4	5	3	0	2
**Multimodal Inseparable (MI)**	12	8	7	8	11	1	0	0
**Unimodal (U)**	12	3	3	7	8	2	0	1
**Multimodal (M)**	18	12	10	12	16	4	0	2
**Separable (S)**	8	4	3	5	6	3	0	2
**Inseparable (I)**	22	11	10	14	18	3	0	1

#### Analysis of significance (inter-relation analysis)

In the first step, the Lilliefors test is used to examine the parametric nature of the results. Subsequently, the Anova and Kruskal-Wallis tests are utilized in order to assess the statistical significance of any findings: the Anova test is used if the data is parametric and the Kruskal-Wallis test is utilized if the data is non-parametric. The results indicated a lack of significance among algorithms (*p = 0*.*4116 > 0*.*05*), benchmark functions (*p = 0*.*4405 > 0*.*05*), and benchmark function characteristics (*p = 0*.*1239 > 0*.*05*). The inter-relation significance analysis between benchmark functions’ characteristics and benchmark functions also shows no significance *(p = 0*.*1767 > 0*.*05)*.

Given the superiority of DE and PSO compared with other approaches considered in this study, further assessment is performed on these two approaches in experiments 2 and 3. In experiment 2 the overall performances of four well-known variations of DE algorithm are assessed against the basic DE. The rationale behind this is to investigate the potential of these modified versions of DE and the possibility of achieving better overall performance. This issue is assessed using a subset of benchmark functions considered in experiment 1 and the experimental results are taken from literature. These benchmark functions include Sphere (US), Rosenbrock (UI), Schwefel and Griewank (MI), and Rastrigin and Michalewicz5 (MS). Similarly, in experiment 3, four well-known variations of PSO are assessed against basic PSO.

### Performance Evaluation on Benchmark Functions between Several Variants of DE

In this comparison, four modified DE-based algorithms have been selected and their performance on a sub-selection of benchmark functions utilized in experiment 1 are evaluated. The selected modified DE-based algorithms include Strategy Adaption Differential Evolution (SADE) [132], Adaptive Differential Evolution with Optional External Archive (JADE) [133], Opposition-based Differential Evolution (OBDE) [134], and Compact Differential Evolution (cDE) [135]. In order to facilitate better understanding of the results with respect to what is reported in experiment 1, the reported results in experiment 1 for original DE and the best performing algorithm are also included. The parameter settings of each of the algorithms can be found in [132–135]. The results are reported in Table 10. SADE, JADE and cDE demonstrated better performance in Sphere function and achieved the theoretical optimum which DE has not been able to achieve in experiment 1. As mentioned before, Sphere is a unimodal and separable function while Rosenbrock is a unimodal and inseparable function. SADE also performed better than all other variations of DE on the Rosenbrock function achieving the theoretical optimum while also outperforming the best performing algorithm on this function in experiment 1 (DE). SADE also performed best among the DE variations on the Schwefel function (multimodal and separable), and also outperformed experiment 1’s champion (ACO), and managed to reach the theoretical optimum. The Rastrigin and Michalewicz5 functions share the same characteristics by being multimodal and separable. In this function, basic DE from literature (see the S1 File) has managed to outperform all DE variant. The overall results presented in Table 10 indicated SADE as the best performing variation of DE among those considered in this experiment.

**Table 10:** Comparison of various DE-based algorithms (Mean ± SD).

Function	Basic DE [140,141]	Strategy Adaptive Differential Evolution (SADE) [132]	Adaptive Differential Evolution with Optional External Archive (JADE) [133]	Opposition-based Differential Evolution (OBDE) [134]	Compact Differential Evolution (cDE) [135]	The best achieved performance in experiment 1
**Sphere**	2.000E-03	**0.000E+00**	**0.000E+00**	5.951E-05	**0.000E+00**	(DE) 5.5942E+03
	±3.000E-03	**±0.000E+00**	**±0.000E+00**	±2.780E-05	**±0.000E+00**	±1.5091E+03
**Rosenbrock**	1.685E+02	**0.000E+00**	1.030E+06	5.362E+01	1.291E+02	(DE) 3.8901E+06
	±6.468E+01	**±0.000E+00**	±0.000E+00	±3.585E+01	±1.83E+02	±2.2417E+06
**Schwefel**	1.027E+04	**0.000E+00**	2.880E+01	-	3.779E+03	(ACO) 3.2250E+03
	±5.218E+02	**±0.000E+00**	±0.000E+00	-	±1.84E+03	±4.5211E+02
**Rastrigin**	1.172E+01	**2.198E-02**	4.700E+02	5.150E+01	7.943E+01	(GA) 5.5900E+01
	±2.538E+00	**±0.000E+00**	±0.000E+00	±1.155E+01	±1.490E+01	±1.4294E+01
**Michalewicz5**	**-4.683E+00**	-4.693E+00	1.470E+02	-4.1054E+00	-4.937E+01	(DE) 4.1803E+00
	**±1.252E-02**	±0.000E+00	±0.000E+00	±4.790E+00	±3.530E+00	±4.2335E-01
**Griewank**	**1.479E-03**	1.724E-02	2.320E+02	1.429E-02	4.982E+03	(DE) 1.1282E+00
	**±2.958E-03**	±0.000E+00	±0.000E+00	±1.850E-02	±3.790E+03	±4.0468E-02

### Performance Evaluation on Benchmark Functions between Several Variants of PSO

Similar to experiment 2, four well-known variations of PSO have been evaluated against the basic PSO and the best performing algorithm found in experiment 1. These selected approaches include Selection PSO (SPSO) [136], Compact PSO [137], Intelligence Single PSO (ISPSO) [138], and Comprehensive Learning PSO (CLPSO) [139]. Table 11 depicts the performance achieved by these selected variations of PSO based on their outcome on the Sphere, Rosenbrock, Schwefel, Rastrigin, Michalewicz5 and Griewank functions. SPSO and ISPOS perform better in the Sphere function and achieve the theoretical optimum and also outperform the result achieved by DE in experiment 1. SPSO has outperformed other variants of PSO and experiment 1’s champion (DE) in Rosenbrock function even it did not managed to obtain the theoretical value. ISPSO demonstrated the best minimization for the Schwefel function in comparison to experiment 1’s champion (ACO) and other variations of PSO. Within Rastrigin function, neither of the PSO variations managed to achieve theoretical optimal value but CLSPO achieved better performance compare to the others. The experiment 1’s champion (DE) has managed to outperform all PSO variants on the Michalewicz5 function, including the basic PSO result obtained from literature (see the S1 File). SPSO is the best performing variation of PSO on the Griewank function and also managed to outperform experiment 1’s champion (DE). The results reported in Table 7 indicate that SPSO is the best performing algorithm among the considered variations of PSO since it is selected as the best PSO variation in 3 out of 6 benchmark functions in addition to being able to outperform the best performing approach in experiment 1 in 3 of the benchmark functions (Sphere, Rosenbrock and Griewank). ISPSO is the second best performing variation of PSO by outperforming other variations in 2 benchmark functions and also outperformed experiment 1’s champion (Sphere and Schwefel). CLPSO is the third best performance where it managed to outperform other competitors in Rastrigin function. cPSO is the least favourable variation among the selected methods because of incompetency to outperform others in any function listed.

**Table 11:** Comparison between various PSO-based algorithms (Mean ± SD).

Function	Basic PSO [140,141]	Selection PSO (SPSO) [136]	Compact PSO (cPSO) [137]	Intelligence Single PSO (ISPSO) [138]	Comprehensive Learning PSO (CLPSO) [139]	The best achieved performance in experiment 1
**Sphere**	**0.000E+00**	**0.000E+00**	6.471E+01	**0.000E+00**	2.870E+03	(DE) 5.5942E+03
	**±0.000E+00**	**±0.000E+00**	±2.280E+01	**±0.000E+00**	±7.443E+02	±1.5091E+03
**Rosenbrock**	6.768E+01	**1.213E+01**	1.291E+02	2.030E+02	5.190E+01	(DE) 3.8901E+06
	±3.037E+01	**±3.533E+01**	±1.830E+02	±3.200E+02	±2.770E+01	±2.2417E+06
**Schwefel**	-6.910E+03	2.560E+03	1.672E+03	**1.183E+01**	-1.080E+04	(ACO) 3.2250E+03
	±4.580E+02	±2.400E+03	±4.49E+02	**±5.900E+00**	±3.610E+02	±4.5211E+02
**Rastrigin**	2.781E+01	1.360E+02	7.943E+01	2.547E+02	**5.610E-06**	(GA) 5.5900E+0
	±7.412E+00	±3.233E+01	±1.490E+01	±4.220E+01	**±4.960E-06**	±1.4294E+01
**Michalewicz5**	-2.491E+00	-	-3.346E+01	-	6.470E-09	**(DE) -4.1803E+00**
	±2.570E-01	-	±1.860E+00	-	±2.320E-09	**±4.2335E-01**
**Griewank**	2.326E-01	**3.913E-03**	4.288E-03	1.123E+01	1.800E-02	(DE) 1.1282E+00
	±9.442E-02	**±1.000E+01**	±1.370E-02	±1.750E+01	±2.060E-02	±4.0468E-02

## Conclusions

This study was concerned with overall performance of various Swarm Intelligence (SI) based approaches and aimed to provide a comparison among the well-known SI-based approaches. A set of methods including Genetic algorithm (GA), Ant Colony Optimization (ACO), Particle Swarm Optimization (PSO), Differential Evolution (DE), Artificial Bee Colony (ABC), Glowworm Swarm Optimization (GSO), and Cuckoo Search Algorithm (CSA) are considered and a selection of thirty benchmark functions that have been utilized in MATLAB to measure the performance of these approaches. These benchmark functions cover a range of characteristics including unimodal, multimodal, separable, and inseparable. The results indicated the superiority of DE with the ability to outperform or perform equally to the best algorithm in 24 out of 30 functions. DE performed very well on multimodal functions, being selected as the best performing approach in 11 out of 12 such functions. This performance repeater in unimodal and inseparable functions in which DE outperformed others in 8 out of 12 and 18 out of 22 functions respectively. PSO is the second best approach that outperformed or performed equally to the best algorithm in 18 out of 30 functions and follows by GA with 14 out of 30. Two extra experiments are offered to capture the performance of four well-known modified versions of PSO and DE on a sub set of 6 benchmark functions. These variations included Strategy Adaption Differential Evolution (SADE) [132], Adaptive Differential Evolution with Optional External Archive (133) [134], Opposition-based Differential Evolution (OBDE) [88], Compact Differential Evolution (cDE) [135], Selection PSO (SPSO) [136], Compact PSO [137], Intelligence Single PSO (ISPSO) [138], and Comprehensive Learning PSO (CLPSO) [139]. The results identified SADE and SPSO as the best performing approaches.

## Supporting Information

S1 PRISMA Checklist (DOCX)

S1 File Supporting text and tables. Table A. Parameter setting utilized by literature studies for each algorithm. Table B. Benchmark Functions Selected for Comparison. Table C. Benchmark Functions Comparison between Several Optimization Techniques (Mean ± SD). Table D. Continued Benchmark Functions Comparison between Several Optimization Techniques (Mean ± SD). Table E. Continued Benchmark Functions Comparison between Several Optimization Techniques (Mean ± SD). Table F. Continued Benchmark Functions Comparison between Several Optimization Techniques (Mean ± SD). Table G. Performance breakdown based on the benchmark functions’ characteristics. Table H. Benchmark Functions Comparison with an offset of mean error (Mean ± SD) and time (Seconds) on several Optimization Techniques. Table I. Benchmark Functions Comparison with an offset of mean error (Mean ± SD) and time (Seconds) on several Optimization Techniques. Table J. Benchmark Functions Comparison with an offset of mean error (Mean ± SD) and time (Seconds) on several Optimization Techniques. Table K. Benchmark Functions Comparison with an offset of mean error (Mean ± SD) and time (Seconds) on several Optimization Techniques. Table L. Benchmark Functions Comparison with an offset of mean error (Mean ± SD) and time (Seconds) on several Optimization Techniques. Table M. Benchmark Functions Comparison with an offset of mean error (Mean ± SD) and time (Seconds) on several Optimization Techniques. Table N. Comparison of various DE-based algorithms (Mean ± SD).
(DOCX)
