# Supplementary material for: A Comprehensive Review of Swarm Optimization Algorithms
Source: PLoS One. 2015 May 18;10(5):e0122827. doi: 10.1371/journal.pone.0122827 (PMC4436220; doi:10.1371/journal.pone.0122827)
Supplement: S1 PRISMA Checklist — (DOCX) [file pone.0122827.s001.docx]

Dear Tara Luckau,

Please find bellow our response to the comments provided.

 Currently, Table 2 still contains hard returns, primarily in the ‘Range’ column. Can you kindly remove these hard returns? Pease recall that these hard returns may have unwanted effects when published. If you want content to be on separate lines, please create a new table row; if you want the content to be in the same cell, please use one space.

Hard returns in Table 2 have been removed.

- Tables must be cited in numerical order on first mention in the main text. Currently, only Table 3 is cited in the paragraph following the heading “Table 3 and 4”. Please cite Table 4 in this section as well.

Table 4 has been cited in the text.

- Currently your inventory of Supporting Information files includes the following: S1-S14 Table, S1 File, Supplement. It appears that S1 File and your Supplement.docx file contain the same content, with the exception that Supplement.docx includes legends/captions for your supporting tables within the text. Please note that we recommend the following changes to your supporting information so that they conform to our requirements:

(1) Please provide only one supporting text document, rather than two supporting files with duplicate information. Only “S1 File” should remain in your inventory.

(2) Please integrate your supporting tables (S1-S14) into “S1 File”, so that each supporting table appears within this supporting file. Each table can appear below the corresponding table legend in the text, for example. This way, readers can access all supporting content within one file. Please use letters to name these tables, rather than the S-numbers (i.e. Tables A-M)

(3) References: please note that all references listed in the “References” section of the main article file must be cited in the main article text. Please remove these references from the References section of the main article, and instead create a new "References" section at the end of S1 File. These references can be renumbered (i.e. starting with "1"), and all citations in the S1 File should be updated accordingly.

(4) Currently you have cited your supporting information in the article text as "the supplement section" or "the supplement". Please use "S1 File" for correct hyperlinking in the final published article.

(5) In the "Supporting Information Legends" section of your main article file, please list “S1 File” only. You may keep the individual legends for your supporting tables here if you wish, but please note that these will be rolled into the legend for S1 File. For example, after these changes, you will have one supporting file (“S1 File”, which includes your supporting text, Ttables A-M, and supporting references 1-16), and your revised Supporting Information Legend section might look like this: "S1 File. Supporting text and tables. Table A. Parameter setting utilized by literature studies for each algorithm. Table B. Benchmark Functions Selected for Comparison Table C. Benchmark Functions ..."

All suggested comments have been answered accordingly. Thank you. Kindly let me know if there is another amendment needed to optimize this paper.
