# Supplementary material for: A Comprehensive Review of Swarm Optimization Algorithms
Source: PLoS One. 2015 May 18;10(5):e0122827. doi: 10.1371/journal.pone.0122827 (PMC4436220; doi:10.1371/journal.pone.0122827)
Supplement: S1 File — Table A. Parameter setting utilized by literature studies for each algorithm. Table B. Benchmark Functions Selected for Comparison. Table C. Benchmark Functions Comparison between Several Optimization Techniques (Mean ± SD). Table D. Continued Benchmark Functions Comparison between Several Optimization Techniques (Mean ± SD). Table E. Continued Benchmark Functions Comparison between Several Optimization Techniques (Mean ± SD). Table F. Continued Benchmark Functions Comparison between Several Optimization Techniques (Mean ± SD). Table G. Performance breakdown based on the benchmark functions’ characteristics. Table H. Benchmark Functions Comparison with an offset of mean error (Mean ± SD) and time (Seconds) on several Optimization Techniques. Table I. Benchmark Functions Comparison with an offset of mean error (Mean ± SD) and time (Seconds) on several Optimization Techniques. Table J. Benchmark Functions Comparison with an offset of mean error (Mean ± SD) and time (Seconds) on several Optimization Techniques. Table K. Benchmark Functions Comparison with an offset of mean error (Mean ± SD) and time (Seconds) on several Optimization Techniques. Table L. Benchmark Functions Comparison with an offset of mean error (Mean ± SD) and time (Seconds) on several Optimization Techniques. Table M. Benchmark Functions Comparison with an offset of mean error (Mean ± SD) and time (Seconds) on several Optimization Techniques. Table N. Comparison of various DE-based algorithms (Mean ± SD). (DOCX) [file pone.0122827.s002.docx]

**Supporting Section for A Comprehensive Review of Swarm Optimization Algorithms**

This section offers three further assessments in order to complement the provided experiments and discussion in the main body of the article. The first compliments assessment captures the performance comparison between all seven selected SI-based algorithms on twenty benchmark functions. This is similar to the first experiment in the manuscript apart from the results are gathered from several literature papers. The second experiment is designed to measure the behaviour of each algorithm when an offset value is added to the global optimum of the benchmark function. The third experiment is the comparison between more advance algorithms with the best performing algorithm from experiments in the main manuscript. More details about the experiments are discussed later in this supplement.

The details of configurations and settings of SI-based approaches reported in these supplement tables are presented in Table A. It is noteworthy that since various settings are utilized within literature for each approach and since the reported results are extracted from various sources in each benchmark function, the reported configurations in Table A are ordered based on their contributing references.

**Table A. Parameter setting utilized by literature studies for each algorithm.**

| **Reference** | **Settings Details** |
| --- | --- |
| [1] | For GA, a binary coded standard GA with 0.8 single point crossover operations are used and the mutation rate is set to 0.01. Stochastic uniform technique is utilized as the selection method and generation gap value is set to 0.9. For DE, *F* is set to 0.5 and crossover value rate is 0.9. For PSO settings, both cognitive and social components are set to 1.8 with inertia weight is set to 0.6. For ABC, only one control parameter is set which is *limit* with depending on *SN* (the number of employed bee or food source) and *D* (the dimension of the problem). |
| [2] | Type of DE used is *DE/rand/1/bin* with *F* is set to 0.9 and crossover rate is set to 0.5 For PSO, the parameter settings used are 1.8 for both acceleration coefficient (social and cognitive) with random value between 0.5 and 1 is utilized for inertia weight. The same approaches in [1] is used for ABC where the *limit* is set by multiplying *SN* and *D*. |
| [3] | The luciferin decay constant, ρ is set to 0.4 with luciferin enhancement constant, γ is set to 0.6. β is a constant parameter which is set to 0.08. Number of neighbours considered is represented by n_t_ and is set to 5. The step size, *s* is set to 0.3. |
| [4] | Parameter utilized considering 10 ants for global search and 30 ants for local search. The pheromone evaporation rate, *p* is set to 0.9. Number of iterations used is 1000 and have been repeated for 20 times. |
| [5] | The same approaches in [1] is used for ABC where the *limit* is set by multiplying *SN* and *D*. |
| [6] | A real constant *F* that controls the differential variation between two solutions is set to 0.5. The change of population size is controlled by value of crossover rate (set to 0.9). This setting is proposed by Goudos et al [6]. |
| [7] | The inertia weight value, ω is set to 0.6 with *C_1_* and *C_2_* share the same value which is 2.0. |
| [8] | The original parameter settings proposed by Ting-En, Jao-Hong, and Lai-Lin [85] and Krihnanand and Ghose [89, 90]. The population size is set to one hundred and the maximum iteration is set to two hundreds. |
| [9] | Scale factor (β) is set to 1.5 and mutation probability value (p_0_) is set to 0.25 following Yang and Deb [102]. |
| [10] | The luciferin decay constant, ρ is set to 0.8 with luciferin enhancement constant, γ is set to 0.05. β is a constant parameter which is set to 0.65. Number of neighbours considered is represented by n_t_ and is set to 5. The step size, *s* is set to 0.5. |

**First Assessment: Performance Evaluation on Benchmark Functions**

In this experiment, the performance of the best optimization techniques from supplement table is compared to several other notable variant or new evolutionary algorithms and assessed based on their overall achieved fitness with variety of benchmark functions of the supplement (same as experiment 1 in the main manuscript, see Table B). If the mean value is less than 1.000E-10, then the result is reported as 0.000E+00. These results are noted in Tables C, D, E, and F in the format of the achieved mean result (Mean) and the standard deviation (SD). It should also be noted that only basic versions of the techniques are considered and no modifications are applied. For better understanding of the results the discussion is divided to three categories of Tables C and D, Tables E and F, and Benchmark function characteristics. This categorization is based on fitness performance with benchmark functions reported in these supplement tables. The square bracket next to the algorithms’ name indicate the references where the results are collected.

**Table B. Benchmark Functions Selected for Comparison**

| No | Function | Formula | Value | Dim | Range | Properties |
| --- | --- | --- | --- | --- | --- | --- |
| 1 | Sumsquare | $f\left(x\right)=\sum_{i=1}^{n} ix_{i}^{2}$ | 0 | 30 | [-5.12, 5.12] | Unimodal, Separable |
| 2 | Sphere | $f\left(x\right)=\sum_{i=1}^{n} x_{i}^{2}$ | 0 | 30 | [-100, 100] | Unimodal, Separable |
| 3 | Quartic | $f\left(x\right)=\sum_{i=1}^{n} ix_{i}^{4}+random\left[0, 1\right)$ | 0 | 30 | [-1.28, 1.28] | Unimodal, Inseparable |
| 4 | Colville | $\begin{matrix} f\left(x\right)=100(x_{1}^{2}-x_{2})^{2}+(x_{1}-1)^{2} \\ +(x_{3}-1)^{2}+90(x_{3}^{2}-x_{4})^{2} \\ +10.1((x_{2}-1)^{2}+\left(x_{4}-1)^{2}\right) \\ +19.8\left(x_{2}-1\right)\left(x_{4}-1\right) \end{matrix}$ | 0 | 4 | [-10, -10] | Unimodal, Inseparable |
| 5 | Powell | $\begin{matrix} f(x)=\sum_{(i=1)}^{(n/k)}(x_{(4i-3)}+10x_{(4i-2)})^{2} \\ +5(x_{(4i-1)}+x_{4}i)^{2}+(x_{(4i-2)}+x_{(4i-1)})^{4} \\ +10(x_{(4i-3)}+x_{4}i)^{4} \end{matrix}$ | 0 | 24 | [-5, 5] | Unimodal, Inseparable |
| 6 | Rosenbrock | $f\left(x\right)=\sum_{i=1}^{n-1} [100(x_{i+1}-x_{i}^{2})^{2}+(x_{i}-1)^{2}]$ | 0 | 30 | [-30, 30] | Unimodal, Inseparable |
| 7 | Dixon-Price | $f\left(x\right)=-\left(x_{1}-1\right)^{2}+\sum_{i=0}^{n} i(2x_{i}^{2}-x_{i}-1)^{2}$ | 0 | 2 | [-100, 100] | Unimodal, Inseparable |
| 8 | Easom | $\begin{matrix} f\left(x\right)=-cos\left(x_{1}\right)cos\left(x_{2}\right) \\ \text{exp}\left(-\left(x_{1}-\pi\right)^{2}-\left(x_{2}-\pi\right)^{2}\right) \end{matrix}$ | -1 | 2 | [-100, 100] | Unimodal, Inseparable |
| 9 | Schwefel | $f\left(x\right)=\sum_{i=1}^{n} -x_{i}\sin\left(\sqrt{\left\vert x_{i}\right\vert}\right)$ | 0 | 30 | [-500, 500] | Multimodal, Separable |
| 10 | Rastrigin | $f\left(x\right)=\sum_{i=1}^{n} x_{i}^{2}-10\cos\left(2\pi x_{i}\right)+10$ | 0 | 30 | [-5.12, 5.12] | Multimodal, Separable |
| 11 | Michalewicz5 | $f\left(x\right)=-\sum_{i=1}^{n} \sin\left(x_{i}\right)(\sin\left(ix_{i}^{2}/\pi\right))^{2m}$ | -4.688 | 5 | [0, π] | Multimodal, Separable |
| 12 | Foxholes | $f\left(x\right)=\left[\frac{1}{500}+\sum_{j=1}^{25} \frac{1}{j+\sum_{i=1}^{2} (x_{i}-a_{ij})^{6}}\right]-1$ | 0.998 | 2 | [-65.536, 65.536] | Multimodal, Separable |
| 13 | Shekel5 | $f\left(x\right)=-\sum_{i=1}^{5} \left[(x-a_{i}\right)(x-a_{i})^{T}+c_{i}]^{-1}$ | -10.151 | 4 | [0, 10] | Multimodal, Inseparable |
| 14 | Perm | $f\left(x\right)=\sum_{k=1}^{n} \sum_{i=1}^{n} \left(i^{k}+\beta)(x_{i}/i)^{k}-1\right)^{2}$ | 0 | 4 | [-D, D] | Multimodal, Inseparable |
| 15 | Fletcher | $f\left(x\right)=\sum_{i=1}^{p} \left(A_{i}-B_{i}\right)^{2}$ | 0 | 10 | [-3.1416, 3.1416] | Multimodal, Inseparable |
| 16 | Langermann | $\begin{matrix} f(x)=-\sum_{i=1}^{m} c_{i}(exp(-1/\sum_{(j=1)}^{n} (x_{j}-a_{i}j)^{2} \\ cos(\pi\sum_{(j=1)}^{n} (x_{j}-a_{i}j)^{2}))) \end{matrix}$ | 0 | 10 | [0, 10] | Multimodal, Inseparable |
| 17 | Schaffer | $f\left(x\right)=0.5+\frac{\sin^{2}(\sqrt{x_{1}^{2}+x_{2}^{2}})-0.5}{\left(1+0.0001\left(\sqrt{x_{1}^{2}+x_{2}^{2}}\right)\right)^{2}}$ | 0 | 2 | [-100, 100] | Multimodal, Inseparable |
| 18 | Griewank | $f\left(x\right)=\frac{1}{4000}\sum_{i=1}^{n} x_{i}^{2}-\prod_{i=1}^{n} \cos\frac{x_{i}}{\sqrt{i}}+1$ | 0 | 30 | [-600, 600] | Multimodal, Inseparable |
| 19 | Kowalik | $f\left(x\right)=\sum_{i=1}^{11} (a_{i}-\frac{x_{1}\left(b_{i}^{2}+b_{i}x_{2}\right)}{b_{i}^{2}+b_{i}x_{3}+x_{4}})^{2}$ | 0.00031 | 4 | [-5, 5] | Multimodal, Inseparable |
| 20 | FletcherPowell5 | $A_{i}=\sum_{j=1}^{n} (a_{ij}\sin\alpha_{j}+b_{ij}\cos\alpha_{j})$ $B_{i}=\sum_{j=1}^{n} (a_{ij}\sin\beta_{j}+b_{ij}\cos\beta_{j})$ $f\left(x\right)=\sum_{i=1}^{p} \left(A_{i}-B_{i}\right)^{2}$ | 0 | 5 | [-π, π] | Multimodal, Inseparable |

**Tables C and D**

Focusing on C and D Tables, the first two benchmark functions (e.g., Sumsquare and Sphere) are unimodal and separable with a theoretical minimization value of zero. In Sumsquare function, DE, ABC and CSA achieved the best minimization with optimal value of zero; in Sphere function only PSO achieved the theoretical optimal value, with ABC being the second best performing. The next five functions in these supplement tables are unimodal and inseparable (Sphere, Quartic, Colville, Powell, Rosenbrock, Dixon-Price, Easom). The results indicate that PSO achieves better minimization performance compared with the other approaches when applied to the Quartic and Coville functions (with zero being the optimal value). DE is the second best performing approach with the Quartic function while no numerical significance can be seen among the methods (all except PSO) with the Coville function. DE is the best performing method on the Powell function with the mean value of 2.170E-07 followed by PSO with the mean value of 1.100E-06. ACO is the best performing approach on Rosenbrock function with GSO as the second best performing. ABC and CSA achieved the theoretical optimal value with the Dixon-price function while a numerical difference can be observed among PSO and DE as the second best performing and GA and GSO as the worst performing approaches on this function. Except for GSO and CSA all other approaches achieved the theoretical optimum value of -1 with the Easom function. The Schwefel and Rastrigin functions are multimodal and separable. ABC and CSA are respectively the best performing approaches with the Schwefel and Rastrigin functions, and showing numerical significance compared to other algorithms when applied to these two functions. Considering the reported results in Tables C and D, PSO and ABC perform best overall due to being selected as the best approaches with four out of ten functions and reaching to the second best rank in another one or two functions. This is closely followed by DE which is considered as the best performing method in three out of ten functions and being the second best performing approach in two other functions. Among the considered methods, GSO is the worst performing method due to not being the best performing approach in either of the functions. This is closely followed by GA and ACO with being selected as the best performing method in only one out of ten functions. It should be noted that we only managed to find results for the application of ACO on five out of ten functions. It is also noteworthy that based on the significant analysis results presented in Table D, significant difference can only be seen within the results of Colville, Schwefel, and Rastrigin functions within which PSO, ABC, and CSA reached to the best performances respectively.

**Table C. Benchmark Functions Comparison between Several Optimization Techniques (Mean ± SD)**

| **Function** | **GA [1, 2, 5]** | **ACO [4, 11-14]** | **PSO [1, 2, 7]** | **DE [1, 2, 6]** |
| --- | --- | --- | --- | --- |
| **Sumsquare** | 1.480E+02 | - | 6.761E-07 | **0.000E+00** |
|  | ±1.241E+01 | - | ±0.000E+00 | **±0.000E+00** |
| **Sphere** | 1.110E+03 | 1.300E+00 | **0.000E+00** | 2.000E+03 |
|  | ±7.421E+01 | ±0.000E+00 | **±0.000E+00** | ±3.000E+03 |
| **Quartic** | 1.807E+00 | 3.310E+00 | **1.157E-03** | 1.363E-03 |
|  | ±2.711E-02 | ±0.000E+00 | **±2.760E-04** | ±4.170E-04 |
| **Colville** | 1.494E-02 | - | **0.000E+00** | 4.091E-02 |
|  | ±7.364E-03 | - | **±0.000E+00** | ±8.198E-02 |
| **Powell** | 9.704E+00 | - | 1.100E-06 | **2.170E-07** |
|  | ±1.548E+00 | - | ±1.600E-06 | **±1.360E-07** |
| **Rosenbrock** | 1.960E+05 | **1.990E-07** | 6.768E-01 | 1.685E+02 |
|  | ±3.850E+06 | **±1.000E-05** | ±3.037E+01 | ±6.468E+01 |
| **Dixon-Price** | 1.220E+03 | - | 6.667E-03 | 6.667E-03 |
|  | ±2.660E+02 | - | ±0.000E+00 | ±0.000E+00 |
| **Easom** | **−1.000E+00** | - | **−1.000E+00** | **−1.000E+00** |
|  | **±0.000E+00** | - | **±0.000E+00** | **±0.000E+00** |
| **Schwefel** | -1.159E+04 | 3.300E+01 | -6.909E+10 | -1.0266E+04 |
|  | ±9.325E+01 | ±4.000E-01 | ±4.580E+02 | ±5.218E+02 |
| **Rastrigin** | 5.292E+01 | 1.000E+02 | 2.781E+01 | 1.172E+01 |
|  | ±4.564E+00 | ±0.000E+00 | ±7.412E+00 | ±2.538E+00 |

**Table D. Continued Benchmark Functions Comparison between Several Optimization Techniques (Mean ± SD)**

| **Function** | **ABC[1, 2, 5]** | **GSO[3, 10, 15, 16]** | **CSA [2, 9]** | ***p*-value** |
| --- | --- | --- | --- | --- |
| **Sumsquare** | **0.000E+00** | 2.871E-03 | **0.000E+00** | 0.4159 |
|  | **±0.000E+00** | ±0.000E+00 | **±0.000E+00** |  |
| **Sphere** | 3.620E-09 | 3.370E+01 | 5.500E+00 | 0.4232 |
|  | ±5.850E-09 | ±7.200E+01 | ±0.000E-00 |  |
| **Quartic** | 1.560E+01 | 1.300E+03 | 3.002E-02 | 0.4232 |
|  | ±4.650E-02 | ±1.000E+00 | ±4.866E-03 |  |
| **Colville** | 9.297E-02 | 9.322E-02 | 9.297E-02 | 0 |
|  | ±6.628E-02 | ±5.411E-02 | ±6.628E-02 |  |
| **Powell** | 3.134E-03 | - | 3.134E-03 | 0.406 |
|  | ±5.030E-02 | - | ±5.030E-04 |  |
| **Rosenbrock** | 4.551E+00 | 2.825E-04 | 8.877E-02 | 0.4232 |
|  | ±4.880E+00 | ±0.000E+00 | ±7.739E-02 |  |
| **Dixon-Price** | **0.000E+00** | 4.470E+04 | **0.000E+00** | 0.4159 |
|  | **±0.000E+00** | ±2.200E+04 | **±0.000E+00** |  |
| **Easom** | **−1.000E+00** | 5.100E+03 | −3.000E-01 | 0.4159 |
|  | **±0.000E+00** | ±2.100E+03 | ±4.702E-01 |  |
| **Schwefel** | **5.500E-01** | 2.660E+03 | −1.251E+04 | 0 |
|  | **±5.000E-01** | ±2.75E+02 | ±7.640E+01 |  |
| **Rastrigin** | 4.531E-01 | 1.537E-01 | **0.000E+00** | 0 |
|  | ±5.150E-01 | ±3.600E-02 | **±0.000E+00** |  |

**Tables E and F**

The first two functions (Michalewicz5 and Foxholes) are multimodal and separable. The results in Tables E and F indicated that PSO is the best performing approach with Michalewicz5, with having numerical differences with other methods. Neither of the approaches is significantly better than others when applied to the Foxholes function with the understanding that no results have been found for ACO and GSO with this function. The remaining eight functions are considered multimodal and inseparable. The functions that fall in this group include Shekel5, Perm, Fletcher, Langermann, Schaffer, Grewank, Kowalik, and FletcherPowell 5. Considering these functions, PSO, DE, CSA became the best performing approaches each achieving best performance in three out of eight functions. PSO performed best with Shekel5, Fletcher, and Langermann, DE being the best performing with the Perm, Fletcher, and Schaffer functions and CSA performing best with the Schaffer, Griewank, and Kowalik functions. It is noticeable that PSO, DE, and GA reached a mean value of zero with Fletcher function. Similarly, DE, ABC, and CSA reached to mean value of zero with Schaffer function. Within the Shekel5 function, DE shares the same performance with ABC and CSA where all of them got minimum optimal value of -10.1532. DE has outperformed other methods when applied to Perm functions with a value of 0.24 which is closest to the optimal value of zero. DE once again shows better performance achieving an optimal minimization value of zero, sharing the same value with GA and PSO, for Fletcher functions. PSO shows the best minimization value in the Langermann functions by achieving an optimal value of zero. With Schaffer functions, DE, ABC and CSA have achieved the optimal value of zero. CSA beats other methods in the Griewank function and also got the optimal minimization value of zero. The Kowalik benchmark function has theoretical optimal value of 0.00031. The best performance with this function is achieved by ABC and CSA with the value of 0.0004266. The FletcherPowell5 function, with an optimal value of zero, is the last function investigated in this study. None of the methods achieved this optimal value with the best performing approach for this function being GA with the value of 0.004303. It is also noteworthy that based on the significant analysis results presented in Table F, significant different can only be seen within the results of the Langermann function within which PSO achieved the best performance.

**Table E. Continued Benchmark Functions Comparison between Several Optimization Techniques (Mean ± SD)**

| **Function** | **GA [1, 2, 5]** | **ACO [4, 11-14]** | **PSO[1, 2, 7]** | **DE[1, 2, 6]** |
| --- | --- | --- | --- | --- |
| **Michalewicz5** | -4.645E+00 | - | -2.491E+00 | -4.683E+00 |
|  | ±9.785E-02 | - | ±2.570E-01 | ±1.253E-02 |
| **Foxholes** | **9.980E-01** | 5.000E+02 | **9.980E-01** | **9.980E-01** |
|  | **±0.000E+00** | ±2.930E+04 | **±0.000E+00** | **±0.000E+00** |
| **Shekel5** | -5.661E+00 | - | **-2.087E+00** | -1.015E+01 |
|  | ±3.867E+00 | - | **±1.178E+00** | ±0.000E+00 |
| **Perm** | 3.027E-01 | - | 3.605E-02 | **2.401E-02** |
|  | ±1.933E-01 | - | ±4.893E-02 | **±4.603E-02** |
| **Fletcher** | **0.000E+00** | - | **0.000E+00** | **0.000E+00** |
|  | **±0.000E+00** | - | **±0.000E+00** | **±0.000E+00** |
| **Langermann** | -9.684E-01 | - | **0.000E+00** | -1.281E+00 |
|  | ±2.875E-01 | - | **±0.000E+00** | ±3.551E-01 |
| **Schaffer** | 4.239E-03 | - | 8.760E-03 | **0.000E+00** |
|  | ±4.239E-03 | - | ±0.000E+00 | **±0.000E+00** |
| **Griewank** | 1.063E+01 | - | 9.227E-03 | 1.108E-03 |
|  | ±1.161E+00 | - | ±1.056E-02 | ±3.440E-03 |
| **Kowalik** | 5.615E-03 | - | 4.906E-04 | 4.266E-04 |
|  | ±8.171E-03 | - | ±3.660E-03 | ±2.730E-03 |
| **FletcherPowell5** | **4.303E-03** | 1.001E+01 | 1.457E+03 | 5.989E+00 |
|  | **±9.469E-03** | ±5.237E+01 | ±1.269E+03 | ±7.334E+00 |

**Table F. Continued Benchmark Functions Comparison between Several Optimization Techniques (Mean ± SD)**

| **Function** | **ABC[1, 2, 5]** | **GSO [3, 10, 15, 16]** | **CSA [2, 9]** | ***p*-value** |
| --- | --- | --- | --- | --- |
| **Michalewicz5** | **-4.688E+00** | 8.000E+03 | **-4.688E+00** | 0.4159 |
|  | **±0.000E+00** | ±3.200E+00 | **±0.000E+00** |  |
| **Foxholes** | **9.980E-01** | - | **9.980E-01** | 0.406 |
|  | **±0.000E+00** | - | **±0.000E+00** |  |
| **Shekel5** | -1.015E+01 | - | -1.015E+01 | 0.406 |
|  | ±0.000E+00 | - | ±0.000E+00 |  |
| **Perm** | 4.111E-02 | - | 4.111E-02 | 0.406 |
|  | ±2.306E-02 | - | ±2.306E-02 |  |
| **Fletcher** | 2.246E+00 | - | 1.186E-02 | 0.406 |
|  | ±1.430E+00 | - | ±5.065E-02 |  |
| **Langermann** | -1.022E+00 | - | -1.403E+00 | 0 |
|  | ±3.223E-01 | - | ±2.378E-01 |  |
| **Schaffer** | **0.000E+00** | 9.322E-05 | **0.000E+00** | 0.4232 |
|  | **±0.000E+00** | ±0.000E+00 | **±0.000E+00** |  |
| **Griewank** | 3.810E-03 | 2.157E-01 | **0.000E+00** | 0.4232 |
|  | ±8.450E-03 | ±1.800E-02 | **±0.000E+00** |  |
| **Kowalik** | **4.266E-04** | - | **4.266E-04** | 0.406 |
|  | **±6.040E-05** | - | **±6.040E-05** |  |
| **FletcherPowell5** | 1.735E-01 | - | 6.820E+00 | 0.4159 |
|  | ±6.810E-02 | - | ±3.785E+01 |  |

**Benchmark functions’ characteristics**

The results presented in Tables C to F can also be investigated based on the characteristics of the fitness functions utilized in the study. Considering categories of i) Unimodal and Separable (US), ii) Unimodal and Inseparable (UI), iii) Multimodal and Separable (MS), iv) Multimodal and Inseparable (MI), v) Multimodal (M), vi) Unimodal (U), vii) Separable (S), and viii) Inseparable (I), Table G is formed. Considering the results presented in G, PSO, ABC and CSA seem to be the best overall performing approach, outperforming other methods in 8 out of 20 functions followed by DE which reached to the best performance in 7 out of 20 functions. Focusing on the breakdown results it is noticeable that PSO has been the best performing method in all categories. It is also striking that GSO has never been the best performing approach. This is with the understanding that only partial results for ACO and GSO is presented here since, no example of them being applied to the missing functions have been found in other studies.

| **Table G. Performance breakdown based on the benchmark functions’ characteristics** | | | | | | | | | |
| --- | --- | --- | --- | --- | --- | --- | --- | --- | --- |
| **Category** | **Number of functions** | **GA** | **ACO** | ***PSO*** | **DE** | **ABC** | **GSO** | **CSA** |  |
| **Being best performing method** | 20 | 4 | 1 | **8** | 7 | **8** | 0 | **8** |  |
| **Unimodal Separable (US)** | 2 | 0 | 0 | **1** | **1** | **1** | 0 | 1 |  |
| **Unimodal Inseparable (UI)** | 6 | 1 | 1 | **3** | 2 | 2 | 0 | 1 |  |
| **Multimodal Separable (MS)** | 4 | 1 | 0 | **1** | 1 | **3** | 0 | **3** |  |
| **Multimodal Inseparable (MI)** | 8 | 2 | 0 | **3** | **3** | 2 | 0 | **3** |  |
| **Unimodal (U)** | 8 | 1 | 1 | **4** | 3 | 3 | 0 | 2 |  |
| **Multimodal (M)** | 12 | 3 | 0 | 4 | 4 | 4 | 0 | **5** |  |
| **Separable (S)** | 6 | 1 | 0 | 2 | 2 | **3** | 0 | **3** |  |
| **Inseparable (I)** | 14 | 3 | 1 | **6** | 5 | 4 | 0 | 4 |  |

**Analysis of significance (inter-relation analysis)**

In the first instance, the Lilliefors test is used to examine the parametric nature of results. Subsequently, the Anova and Kruskal-Wallis tests are utilized in order to assess the statistical significance of any findings: the Anova test is used if it is parametric and if it is non-parametric, the Kruskal-Wallis test is utilized. ACO and GSO are omitted in the significant analysis since only partial results are available for them. It should be noted that the presented results in Tables C to F only represent the mean value of multiple execution of algorithms and therefore the current study lacks sufficient data points in order to assess inter-relation significant analysis among factors such as algorithms, benchmark functions, and benchmark functions’ characteristics. The results indicated a lack of significance among algorithms (*p=0.4246 > 0.05*), benchmark functions (*p=0.4045 > 0.05*), and benchmark function characteristics (*p=0.1639 > 0.05*). The inter-relation significance analysis between benchmark functions’ characteristics and benchmark functions also shows no significance *(p=0.1767 > 0.05)*. In addition to overall analysis of significance, this analysis is also applied to individual benchmark functions capturing the existent of significance among algorithms applied to that function. These results are illustrated as an additional column in Tables D and E. The results indicated a lack of significant difference in results achieved with various evolutionary methods on most benchmark functions except for Colville (UI), Schwefel (MS), Rastrigin (MS), and Langermann (MI) *(p=0 < 0.05)*. PSO is marked as the best performing approach on the Colville and Langermann functions and ABC and CSA are the best techniques on the Schwefel and Rastrigin functions respectively.

Given the superiority of PSO and DE compared with other approaches considered in this study, further assessment is performed on these two approaches in experiments 2 and 3. In experiment 2 the overall performances of four well-known variations of DE algorithms are assessed against the basic DE. The rationale behind this is to investigate the potential of these modified versions of DE and the possibility of achieving better overall performance. This issue is assessed using a subset of benchmark functions considered in experiment 1 for which experimental results with these algorithms are found. These benchmark functions include Sphere (US), Rosenbrock (UI), Schwefel and Griewank (MI), and Rastrigin and Michalewicz5 (MS). Similarly, in experiment 3, four well-known variations of PSO are assessed against basic PSO.

**Second Assessment: Performance Evaluation on Benchmark Functions with an offset added**

In this assessment, the experiment done in the main paper is evaluated again with adding an offset value to the theoretical value of each benchmark. The aim for this experiment is to assess the behaviour of all algorithms when this offset value is added. Hypothetically, the output of each algorithm should be within the region of new global optima. The experiment uses the same parameter settings for all selected algorithms with the same population size and number of iterations as well. However, this assessment has been executed ten times only and the mean value are reported in Tables H to M. The same benchmark functions are used and time taken to complete the run is taken in to the account too. Focusing on Tables H and I, noted that all the benchmark function listed are unimodal. The results indicate that neither of the algorithms are moving towards the old optimal value. Instead, the result indicate that all of them are performing the minimization within the region of the offset. For instance, in Beale function, PSO managed to achieve the theoretical optimal value which is zero in the experiment done in the main manuscript. In this assessment, PSO also managed to find the theoretical value which is π. In terms of time taken to complete the run, most of the algorithms are taking longer time to complete the execution. However, ABC still become the fastest algorithm to complete the execution on the benchmark functions. Now focusing on Tables J to M, all the benchmarks listed are multimodal with Tables J and K being separable and Tables L and M being inseparable. The same behaviour can be observed from the results in Tables J and K where neither of the algorithms are given any result close to the theoretical value without an offset value. In Rastrigin function, where in the main manuscript GA has become the best performing algorithm, when an offset value is added, all algorithms managed to find the new theoretical optimal value which is π. In Tables L and M, the same behaviour is demonstrated by all algorithms where none of them are producing the result close to theoretical value without an offset value. Therefore, it is the conclusion that neither of these selected algorithms do not have any systematic error when an offset is given to the benchmark function.

| **Table H. Benchmark Functions Comparison with an offset of mean error (Mean ± SD) and time (Seconds) on Several Optimization Techniques** | | | | |
| --- | --- | --- | --- | --- |
| **Function** | **GA** | **ACO** | **PSO** | **DE** |
| Sphere (Separable) | 2.3142E+01 | 2.3342E+01 | 2.3147E+01 | 2.3142E+01 |
|  | ±0.0000E+00 | ±4.2164E-01 | ±1.3002E-02 | ±0.0000E+00 |
|  | (2.8719s) | (2.0297s) | (1.8375s) | (4.5141s) |
| Sumsquare (Separable) | 1.9783E+00 | 1.9783E+00 | 2.2344E+00 | 1.1577E+00 |
|  | ±0.0000E+00 | ±0.0000E+00 | ±5.4463E-01 | ±8.4752E-03 |
|  | (3.0359s) | (2.0313s) | (1.8453s) | (4.3547s) |
| Beale (Inseparable) | 3.8447E+00 | 3.8447E+00 | 3.1416E+00 | 3.4558E+00 |
|  | ±0.0000E+00 | ±0.0000E+00 | ±0.0000E+00 | ±9.9346E-01 |
|  | (3.0422s) | (2.5922s) | (1.8766s) | (8.3203s) |
| Colville (Inseparable) | 3.1416E+00 | 7.7502E+01 | 3.1416E+00 | 4.4813E+00 |
|  | ±0.0000E+00 | ±3.4021E+01 | ±0.0000E+00 | ±1.8383E+00 |
|  | (2.9016s) | (2.5031s) | (1.9875s) | (5.0734s) |
| Dixon-Price (Inseparable) | 4.1416E+00 | 4.1416E+00 | 3.8460E+00 | 3.1416E+00 |
|  | ±0.0000E+00 | ±0.0000E+00 | ±0.0000E+00 | ±0.0000E+00 |
|  | (2.6156s) | (1.9828s) | (1.8156s) | (4.3438s) |
| Easom (Inseparable) | 2.2942E+00 | 2.4570E+00 | 2.6895E+00 | 2.1416E+00 |
|  | ±2.9772E-01 | ±4.1429E-01 | ±3.9740E-01 | ±0.0000E+00 |
|  | (8.5406s) | (5.1422s) | (4.6594s) | (10.7859s) |
| Matyas (Insepearable) | 3.1416E+00 | 3.1416E+00 | 3.1416E+00 | 3.1416E+00 |
|  | ±0.0000E+00 | ±0.0000E+00 | ±0.0000E+00 | ±0.0000E+00 |
|  | (7.2203s) | (5.0141s) | (4.6094s) | (12.1359s) |
| Powell (Inseparable) | 3.9134E+02 | 9.2499E+03 | 9.2165E+03 | 2.6528E+02 |
|  | ±1.4986E+02 | ±1.2785E+03 | ±3.1335E+03 | ±1.6417E+02 |
|  | (9.8125s) | (14.4438s) | (6.4984s) | (30.7656s) |
| Rosenbrock (Inseparables) | 3.3416E+00 | 3.1416E+00 | 3.1416E+00 | 3.3687E+00 |
|  | ±4.2164E-01 | ±0.0000E+00 | ±0.0000E+00 | ±6.9767E-01 |
|  | (2.5359s) | (1.9813s) | (1.8281s) | (4.3969s) |
| Schwefel (Inseparable) | 3.1419E+00 | 3.2751E+00 | 3.1419E+00 | 1.4985E+01 |
|  | ±0.0000E+00 | ±7.2807E-02 | ±0.0000E+00 | ±3.7453E+01 |
|  | (2.6891s) | (2.2125s) | (1.8078s) | (4.3688s) |
| Trid 6 (Inseparable) | -2.7558E+01 | -2.5058E+01 | -4.6858E+01 | -4.5995E+01 |
|  | ±1.2597E+01 | ±7.2388E+00 | ±1.9973E-06 | ±1.7784E+00 |
|  | (8.7219s) | (7.3781s) | (5.3344s) | (13.7109s) |
| Zakharov (Inseparable) | 2.3435E+01 | 7.2729E+01 | 4.4933E+00 | 4.3217E+00 |
|  | ±7.8062E+00 | ±1.6222E+01 | ±1.7149E+00 | ±1.7381E+00 |
|  | (10.3484s) | (10.1938s) | (5.2359s) | (18.4359s) |

| **Table I. Benchmark Functions Comparison with an offset of mean error (Mean ± SD) and time (Seconds) on Several Optimization Techniques** | | | | |
| --- | --- | --- | --- | --- |
| **Function** | **ABC** | **GSO** | **CSA** | ***p*-value** |
| Sphere (Separable) | 1.1667E+05 | 1.1829E+06 | 4.2615E+04 | 0.0001 |
|  | ±8.3508E+03 | ±8.0723E+04 | ±5.5047E+04 |  |
|  | (1.3891s) | (5.8674s) | (1.6746s) |  |
| Sumsquare (Separable) | 1.7476E+01 | 2.0526E+01 | 1.6531E+01 | 0.001 |
|  | ±3.1623E-01 | ±2.9771E-01 | ±7.9493E-01 |  |
|  | (1.7153s) | (8.1047s) | (1.3182s) |  |
| Beale (Inseparable) | 3.1416E+00 | 4.8639E+00 | 3.2073E+00 | 0.0001 |
|  | ±0.0000E+00 | ±6.0540E-02 | ±2.1365E-02 |  |
|  | (1.4084s) | (7.3430s) | (1.7775s) |  |
| Colville (Inseparable) | 7.6902E+01 | 1.2015E+02 | 6.7322E+01 | 0.0001 |
|  | ±2.8049E+01 | ±2.6130E+01 | ±5.5250E+00 |  |
|  | (1.4339s) | (7.7588s) | (1.9777s) |  |
| Dixon-Price (Inseparable) | 4.7048E+00 | 5.3979E+00 | 4.1416E+00 | 0.001 |
|  | ±0.0000E+00 | ±0.0000E+00 | ±0.0000E+00 |  |
|  | (1.6832s) | (5.1531s) | (1.6965s) |  |
| Easom (Inseparable) | 6.2851E+00 | 7.3501E+00 | 6.2814E+00 | 0.001 |
|  | ±1.2470E-04 | ±5.3736E-02 | ±5.6839E-04 |  |
|  | (1.4217s) | (12.7382s) | (1.2498s) |  |
| Matyas (Insepearable) | 3.1416E+00 | 2.4540E+00 | 3.1416E+00 | 0.001 |
|  | ±0.0000E+00 | ±2.6413E-01 | ±0.0000E+00 |  |
|  | (2.0500s) | (10.3101s) | (4.7204s) |  |
| Powell (Inseparable) | 4.3360E+05 | 3.6757E+06 | 1.2345E+04 | 0.001 |
|  | ±2.7039E+05 | ±6.5117E+06 | ±3.7790E+03 |  |
|  | (1.5690s) | (11.5516s) | (1.7003s) |  |
| Rosenbrock (Inseparable) | 4.8807E+10 | 1.7626E+12 | 1.2493E+07 | 0.001 |
|  | ±1.1684E+10 | ±2.7674E+12 | ±8.6725E+06 |  |
|  | (1.4656s) | (15.2344s) | (2.0009s) |  |
| Schwefel (Inseparable) | 3.6619E+03 | 7.7821E+04 | 6.6619E+03 | 0.001 |
|  | ±2.3244E+02 | ±2.0826E+03 | ±4.1047E+02 |  |
|  | (1.4406s) | (8.5055s) | (1.8977s) |  |
| Trid 6 (Inseparable) | -3.0212E+01 | -1.8512E+01 | -3.6512E+01 | 0.001 |
|  | ±2.8206E+00 | ±1.1836E+01 | ±3.2813E+00 |  |
|  | (1.5001s) | (14.1288s) | (4.7548s) |  |
| Zakharov (Inseparable) | 8.3967E+01 | 1.2387E+02 | 9.7332E+00 | 0.001 |
|  | ±5.5187E+00 | ±1.1658E+01 | ±2.9051E+00 |  |
|  | (1.6347s) | (18.9145s) | (4.6019s) |  |

| **Table J. Benchmark Functions Comparison with an offset of mean error (Mean ± SD) and time (Seconds) on Several Optimization Techniques** | | | | |
| --- | --- | --- | --- | --- |
| **Function** | **GA** | **ACO** | **PSO** | **DE** |
| Bohachecvsky1 (Separable) | 3.1416E+00 | 3.4616E+00 | 3.1461E+00 | 3.1416E+00 |
|  | ±0.0000E+00 | ±6.7462E-01 | ±0.0000E+00 | ±0.0000E+00 |
|  | (6.7969s) | (5.2063s) | (4.8172s) | (10.6219s) |
| Booth (Separable) | 3.1416E+00 | 3.1416E+00 | 3.1416E+00 | 3.1416E+00 |
|  | ±0.0000E+00 | ±0.0000E+00 | ±0.0000E+00 | ±0.0000E+00 |
|  | (6.9844s) | (5.1188s) | (4.7328s) | (10.6797s) |
| Branin (Separable) | 3.6395E+00 | 3.6395E+00 | 3.6395E+00 | 3.5395E+00 |
|  | ±0.0000E+00 | ±0.0000E+00 | ±0.0000E+00 | ±0.0000E+00 |
|  | (7.3234s) | (5.0594s) | (4.6703s) | (9.9766s) |
| Michalewciz5 (Separable) | 1.5765E+00 | 1.5765E+00 | 1.8413E+00 | -1.0807E+00 |
|  | ±0.0000E+00 | ±1.0134E-05 | ±3.5033E-01 | ±4.3276E-01 |
|  | (3.2828s) | (2.7813s) | (1.9734s) | (8.5109s) |
| Rastrigin (Separable) | 3.1416E+00 | 3.1416E+00 | 3.1416E+00 | 3.1416E+00 |
|  | ±0.0000E+00 | ±0.0000E+00 | ±0.0000E+00 | ±0.0000E+00 |
|  | (2.8438s) | (1.9938s) | (1.7938s) | (4.6000s) |
| Shubert (Separable) | -1.2570E+02 | -1.2570E+02 | -1.2570E+02 | -1.8359E+02 |
|  | ±0.0000E+00 | ±0.0000E+00 | ±0.0000E+00 | ±0.0000E+00 |
|  | (7.8453s) | (5.0906s) | (4.6797s) | (10.1031s) |
| Ackley (Inseparable) | 1.9883E+01 | 1.8034E+01 | 2.0102E+01 | 3.5942E+01 |
|  | ±1.3559E+00 | ±8.1736E-01 | ±6.5316E+00 | ±1.4179E+00 |
|  | (11.4172s) | (21.8078s) | (7.1531s) | (16.0318s) |
| Bohachecvsky2 (Inseparable) | 3.1416E+00 | 3.9416E+00 | 3.1437E+00 | 3.1416E+00 |
|  | ±0.0000E+00 | ±8.4327E-01 | ±0.0000E+00 | ±0.0000E+00 |
|  | (6.8016s) | (5.2781s) | (4.6797s) | (10.3938s) |
| Bohachecvsky3 (Inseparable) | 3.1416E+00 | 3.1416E+00 | 3.1416E+00 | 3.1416E+00 |
|  | ±0.0000E+00 | ±0.0000E+00 | ±0.0000E+00 | ±0.0000E+00 |
|  | (6.9969s) | (5.1750s) | (4.6594s) | (10.7781s) |

| **Table K. Benchmark Functions Comparison with an offset of mean error (Mean ± SD) and time (Seconds) on Several Optimization Techniques** | | | | |
| --- | --- | --- | --- | --- |
| **Function** | **ABC** | **GSO** | **CSA** | ***p*-value** |
| Bohachecvsky1 (Separable) | 3.1416E+00 | 4.9101E+00 | 3.1543E+00 | 0.001 |
|  | ±0.0000E+00 | ±8.0414E-02 | ±8.0204E-03 |  |
|  | (1.4695s) | (11.2283s) | (3.0481s) |  |
| Booth (Separable) | 3.1416E+00 | 7.7416E+00 | 3.1416E+00 | 0.001 |
|  | ±0.0000E+00 | ±2.3002E-01 | ±0.0000E+00 |  |
|  | (1.5790s) | (11.8496s) | (3.2089s) |  |
| Branin (Separable) | 3.5395E+00 | 4.0623E+01 | 3.5395E+00 | 0.001 |
|  | ±0.0000E+00 | ±8.6588E-01 | ±0.0000E+00 |  |
|  | (1.4703s) | (10.4698s) | (3.6762s) |  |
| Michalewciz5 (Separable) | -4.2242E-01 | 2.1554E+00 | 1.6024E+00 | 0.001 |
|  | ±3.2433E-02 | ±2.5724E-01 | ±6.7793E-02 |  |
|  | (1.3810s) | (8.2572s) | (1.9797s) |  |
| Rastrigin (Separable) | 3.1416E+00 | 3.1316E+00 | 3.1416E+00 | 0.001 |
|  | ±0.0000E+00 | ±0.0000E+00 | ±0.0000E+00 |  |
|  | (1.6266s) | (6.1075s) | (2.0863s) |  |
| Shubert (Separable) | -1.3113E+02 | -8.9591E+01 | -2.8007E+02 | 0.001 |
|  | ±3.2040E+00 | ±0.0000E+00 | ±2.1783E+01 |  |
|  | (1.4623s) | (6.5500s) | (1.0811s) |  |
| Ackley (Inseparable) | 2.3843E+01 | 2.3125E+01 | 2.0190E+01 | 0.001 |
|  | ±3.7364E-02 | ±5.3322E-01 | ±6.5824E+00 |  |
|  | (1.8859s) | (17.2865s) | (6.5242s) |  |
| Bohachecvsky2 (Inseparable) | 3.6137E+00 | 3.3564E+01 | 8.5660E+00 | 0.001 |
|  | ±2.8564E-01 | ±6.9014E+00 | ±2.6812E+00 |  |
|  | (1.5684s) | (11.8235s) | (3.2652s) |  |
| Bohachecvsky3 (Inseparable) | 3.6639E+00 | 1.5960E+01 | 5.9639E+00 | 0.001 |
|  | ±3.3498E-01 | ±4.6593E-01 | ±4.6749E-01 |  |
|  | (1.4508s) | (11.3620s) | (3.1835s) |  |

| **Table L. Benchmark Functions Comparison with an offset of mean error (Mean ± SD) and time (Seconds) on Several Optimization Techniques** | | | | |
| --- | --- | --- | --- | --- |
| **Function** | **GA** | **ACO** | **PSO** | **DE** |
| Bukin6 (Inseparable) | 3.1416E+00 | 3.1416E+00 | 3.1816E+00 | 3.1728E+00 |
|  | ±0.0000E+00 | ±0.0000E+00 | ±5.1640E-02 | ±4.9062E-02 |
|  | (6.7938s) | (5.0281s) | (4.7203s) | (10.2078s) |
| Drop-Wave (Inseparable) | 2.1416E+00 | 2.1416E+00 | 2.2033E+00 | 2.1416E+00 |
|  | ±0.0000E+00 | ±0.0000E+00 | ±1.6481E-01 | ±0.0000E+00 |
|  | (7.7047s) | (5.0234s) | (4.7328s) | (11.9516s) |
| Egg Holder (Inseparable) | -9.1586E+02 | -8.3021E+02 | -7.6281E+02 | -8.9982E+02 |
|  | ±3.4915E+01 | ±7.1164E+01 | ±1.5226E+02 | ±7.2534E+01 |
|  | (7.7391s) | (5.5984s) | (4.7406s) | (9.8375s) |
| Goldstein-Price (Inseparable) | 6.1416E+00 | 6.1416E+00 | 6.1416E+00 | 6.1416E+00 |
|  | ±0.0000E+00 | ±0.0000E+00 | ±0.0000E+00 | ±0.0000E+00 |
|  | (7.0422s) | (5.2250s) | (4.7313s) | (9.7578s) |
| Griewank (Inseparable) | 3.1459E+00 | 3.1531E+00 | 3.3975E+00 | 3.1465E+00 |
|  | ±3.7608E-03 | ±9.6258E-03 | ±1.7823E-01 | ±4.3493E-03 |
|  | (2.9875s) | (2.2109s) | (1.8234s) | (4.6031s) |
| McCormick (Inseparable) | 1.4210E+00 | 1.4210E+00 | 1.3968E+00 | 1.2694E+00 |
|  | ±0.0000E+00 | ±0.0000E+00 | ±3.2802E-02 | ±5.2815E-02 |
|  | (7.3906s) | (5.0688s) | (4.7375s) | (10.3828s) |
| Perm (Inseparable) | 7.3129E+02 | 7.3129E+02 | 7.3129E+02 | 8.0119E+01 |
|  | ±0.0000E+00 | ±0.0000E+00 | ±0.0000E+00 | ±2.3110E+02 |
|  | (3.2172s) | (2.6906s) | (2.2906s) | (5.8828s) |
| Schaffer 2 (Inseparable) | 3.1436E+00 | 3.1461E+00 | 3.2435E+00 | 3.1416E+00 |
|  | ±2.2820E-03 | ±5.2786E-03 | ±1.4103E-01 | ±0.0000E+00 |
|  | (8.0281s) | (5.0297s) | (4.7031s) | (11.7000s) |
| Schaffer 4 (Inseparable) | 3.6417E+00 | 3.6417E+00 | 3.6417E+00 | 3.6417E+00 |
|  | ±5.6856E-07 | ±4.2642E-07 | ±8.3150E-07 | ±4.6620E-08 |
|  | (7.8484s) | (5.0672s) | (4.6906s) | (10.9594s) |

| **Table M. Benchmark Functions Comparison with an offset of mean error (Mean ± SD) and time (Seconds) on Several Optimization Techniques** | | | | |
| --- | --- | --- | --- | --- |
| **Function** | **ABC** | **GSO** | **CSA** | ***p*-value** |
| Bukin 6 (Inseparable) | 3.1416E+00 | 6.7258E+00 | 3.1421E+00 | 0.001 |
|  | ±0.0000E+00 | ±1.0744E-01 | ±1.7146E-05 |  |
|  | (1.5388s) | (11.3098s) | (3.6373s) |  |
| Drop-Wave (Inseparable) | 6.0183E+00 | 9.5552E+00 | 5.7294E+00 | 0.001 |
|  | ±1.7913E-02 | ±2.6682E-02 | ±2.4066E-02 |  |
|  | (1.5806s) | (11.7843s) | (4.1330s) |  |
| Egg Holder (Inseparable) | -8.8333E+02 | -8.2130E+01 | -8.8333E+02 | 0.001 |
|  | ±5.5959E+01 | ±6.5870E+00 | ±5.5959E+01 |  |
|  | (1.7792s) | (10.6192s) | (3.8207s) |  |
| Goldstein-Price (Inseparable) | 6.1416E+00 | 9.9351E+00 | 6.1416E+00 | 0.001 |
|  | ±0.0000E+00 | ±2.3954E-01 | ±0.0000E+00 |  |
|  | (1.4733s) | (10.7174s) | (4.1399s) |  |
| Griewank (Inseparable) | 3.7294E+01 | 1.0017E+02 | 1.5553E+01 | 0.001 |
|  | ±2.2269E+00 | ±3.0447E+00 | ±3.3997E-01 |  |
|  | (2.3000s) | (13.9829s) | (2.1890s) |  |
| McCormick (Inseparable) | 1.3034E+00 | 4.4223E+00 | 1.3012E+00 | 0.001 |
|  | ±2.3137E-02 | ±1.1802E-01 | ±2.0994E-02 |  |
|  | (1.5655s) | (10.6473s) | (4.1500s) |  |
| Perm (Inseparable) | 9.4345E+02 | 7.3129E+02 | 7.3129E+02 | 0.001 |
|  | ±0.0000E+00 | ±0.0000E+00 | ±0.0000E+00 |  |
|  | (1.8073s) | (7.2672s) | (1.7579s) |  |
| Schaffer 2 (Inseparable) | 3.1557E+00 | 2.0628E+01 | 3.1561E+00 | 0.001 |
|  | ±1.9614E-02 | ±8.5832E-01 | ±1.9321E-02 |  |
|  | (1.5254s) | (11.1953s) | (3.6848s) |  |
| Schaffer 4 (Inseparable) | 3.6576E+00 | 2.1360E+01 | 3.6499E+00 | 0.001 |
|  | ±1.8986E-02 | ±8.4297E-01 | ±8.0204E-03 |  |
|  | (1.5092s) | (11.1791s) | (4.2852s) |  |

**Third Assessment: Performance Evaluation on Benchmark Functions between Advanced Optimization Algorithms**

In this experiment, the performance of the best optimization techniques from supplement table is compared to several advanced optimization algorithms and assessed based on their overall achieved fitness with variety of benchmark functions (same as assessment 1). If the mean value is less than 1.000E-10, then the result is reported as 0.000E+00. These results are noted in Table N in the format of the achieved mean result (Mean) and the standard deviation (SD). Noted that all the result are obtained from several papers and references are available next to the algorithms’ names. The advanced optimization algorithms selected are Evolutionary Strategies with Covariance Matrix Adaptation (CMA-ES) [14], Quantum Evolutionary Algorithm (QEA)[15], Fast Evolutionary Programming (FEP) [120], Hybrid Algorithm between Memetic Algorithm with PSO and DE (pMA_BLX-α) [16], and Grey Wolf Optimization [120]. Five well-known benchmark functions are selected to measure the performance of all these advanced optimization techniques. Table N depicts the performance achieved by these algorithms based on their mean value on the Ackley, Griewank, Rastrigin, Rosenbrock, and Schwefel. GWO has managed to achieve theoretical optimal value which is zero for Ackley function. The second best performance is QEA where it managed to achieve 1.966E-03. Both of these algorithms managed to outperform the best achieved performance in experiment 1 (experiment in main manuscript). The second benchmark functions is Griewank where the best performing algorithm is assessment 1’s champion (CSA) with the result of theoretical optimal value. Hybrid algorithm (pMA_BLX-α) managed to become the second best performing algorithm with the outcome of 1.240E-01. QEA has become the third best with mean value of 1.543E-01. In Rastrigin function, CSA which is assessment 1’s champion has outperform all advanced optimization algorithms by achieving theoretical optimal value. Hybrid Algorithm (pMA_BLX-α) achieved 1.950E-01 mean value and managed to outperform others algorithm in Rosenbrock function. Schwefel function has theoretical optimization value of zero and neither of any algorithm managed to achieve that optimal value but ABC managed to outperform other advanced optimization algorithm with the result of 5.500E-01. The second best performing algorithm is QEA with a mean value of 4.826E+02. The results reported in Table N show that CSA is the best performing algorithm among the considered algorithms since they managed to outperform the others in 2 out of 5 benchmark functions. The second best is GWO, ACO and ABC where they outperformed others once in five benchmark functions.

**Table N. Comparison of various DE-based algorithms (Mean ± SD)**

| **Function** | **CMA-ES (Covariance Matrix Adaption - ES) [16]** | **Quantum Evolutionary Algorithm (QEA)[17]** | **Fast Evolutionary Programming (FEP) [19]** | **Hybrid Algorithm (pMA_BLX-α) [18]** | **Grey Wolf Optimizer (GWO) [19]** | **The best achieved performance in assessment 1** |
| --- | --- | --- | --- | --- | --- | --- |
| Ackley | 3.701E+04 | 1.966E-03 | 1.800E-02 | 6.100E-09 | **0.000E+00** | (*DE) 1.280E+01 |
|  | ±0.000E+00 | ±1.966E-03 | ±2.100E-03 | ±1.980E-08 | **±7.783E-02** | ±8.415E-01 |
| Griewank | 3.111E+03 | 1.543E-01 | 1.600E-02 | 1.240E-01 | 4.485E-03 | **(CSA) 0.000E+00** |
|  | ±0.000E+00 | ±1.251E-01 | ±2.200E-02 | ±7.570E-02 | ±6.659E-03 | **±0.000E+00** |
| Rastrigin | 1.474E+05 | 2.102E+01 | 5.060E+00 | 1.590E-01 | 3.105E-01 | **(CSA) 0.000E+00** |
|  | ±0.000E+00 | ±7.587E+00 | ±5.87E+00 | ±3.720E-01 | ±4.736E+01 | **±0.000E+00** |
| Rosenbrock | - | 1.518E+02 | 1.470E+02 | 1.950E-01 | 2.6813E+01 | **(ACO) 1.990E-07** |
|  | - | ±3.066E+02 | ±0.000E+00 | ±5.750E-10 | ±6.990E+01 | **±1.000E-05** |
| Schwefel | 4.381E+04 | 4.862E+02 | -1.255E+04 | 4.600E+02 | -6.123E+03 | **(ABC) 5.500E-01** |
|  | ±0.000E+00 | ±1.946E+02 | ±5.260E+01 | ±3.490E+02 | ±4.087E+03 | **±5.000E-01** |

*The results are taken from the experiment 1 in the main manuscript since the results from the same benchmark function is not available in the assessment 1.

**Supporting Conclusions**

This supplement provided an extra assessment for swarm intelligence algorithm in the main paper. From the first assessment, the results show that PSO, ABC and CSA share the best performing algorithm as they managed to outperform the others in eight out of twenty benchmark functions. Although, the experiment in the main manuscript indicates ABC and CSA did not perform as well as DE and PSO but they can give a competitive result if the iteration numbers are higher. The second assessment indicates that all these swarm intelligence based algorithm do not have any systematic error when an offset is added into the benchmark functions. The third assessment is focused on comparing the advanced optimization algorithm. The best performing algorithm in assessment 1 is compared to five advanced optimization algorithm. Surprisingly, PSO managed to become the best performing algorithm along with the Hybrid Algorithm (pMA_BLX-α). It indicates that standard PSO is quite a competitive swarm intelligence based algorithm.

**Supporting References**

1. Karaboga D, Akay B, A comparative study of Artificial Bee Colony algorithm. Applied Mathematics and Computation. 2009: 108-132.
2. Civicioglu P, Besdok E, A conceptual comparison of cuckoo-search, particle swarm optimization, differential evolution and artificial bee colony algorithm. Artificial Intelligence Review, Springer. 2013: 315-346.
3. Zhao G, Zhou Y, Wang Y, The Glowworm Swarm Optimization Algorithm with Local Search Operator. Journal of Information & Computational Science. 2012: 1299-1308.
4. Wang X, Gao XZ, Ovaska SJ, A Hybrid Optimization Algorithm Based on Ant Colony and Immune Principles. International Journal of Computer Science & Application. 2007: 30-44.
5. Venkata R, Patel V, An improved teaching-learning-based optimization algorithm for solving unconstrained optimization problems. Transactions D: Computer Science & Engineering and Electrical Engineering. 2013: 710-720.
6. Goudos SK, Baltzis KB, Antoniadis K, Zaharis Z D, Hilas CS, A Comparative Study of Common and Self-Adaptive Differential Evolution Strategies on Numerical Benchmark Problems. Procedia Computer Science. 2011: 83-88.
7. Ghosh S, Das S, Kundu D, Suresh K, Abraham A, Inter-particle communication and search dynamics of lbest partice swarm optimizers: an analysis. Information Science. 2012: 156-168.
8. Niwa J, Glowworm optimization. International Conference on Swarm and Evolutionary Computation. Springer-Verlag. 2012: 310-316.
9. Xu G, An adaptive parameter tuning of particle swarm optimization algorithm. Applied Mathematics and Computation. 2013: 4560-4569.
10. Iranpour B, Meybodi M, An Improved Fuzzy Based Glowworm Algorithm. International Journal of Engineering and Technology. 2012: 900-905.
11. Liao T, Molina D, Stutzle T, Oca MAM, Dorigo M, An ACO algorithm benchmarked on the BBOB noiseless function testbed. International Conference on Genetic and Evolutionary Computation Conference Companion. 2012: 159-166.
12. Liao T, Oca MAM, Aydin D, Stutzle T, Dorigo M, An Incremental Ant Colony Algorithm with Local Search for Continuous Optimization. Genetic and Evolutionary Computation Conference. 2011: 125-132.
13. Socha K, Dorigo M, Ant colony optimization for continuous domains. European Journal of Operational Research. 2008: 1155-1173.
14. Maniezzo V, Gambardella LM, Luigi F, Ant Colony Optimization. 2001: 1-21. Available: http://www.cs.unibo.it/bison/publications/ACO.pdf. Accessed 02 December 2013.
15. Wu B, Qian C, Ni W, Fan S, The improvement of glowworm swarm optimization for continuous optimization problems. Expert Systems with Applications. 2012: 6335-6342.
16. Hansen N, Kern S, Evaluating the CMA Evolution Strategy on Multimodal Test Functions. Parallel Problem Solving from Nature - PPSN VIII. 2004;3242: 282-291.
17. Ma T, Yan Q, Xuan W, Wang B, A Comparative Study of Quantum Evolutionary Algorithm and Particle Swarm Optimization for Numerical Optimization Problems. International Journal of Digital Content Technology and its Applications. 2011;5(7): 182-190.
18. Devi S, Jadhav DG, Pattnaik SS, PSO Based Memetic Algorithm for Unimodal and Multimodal Function Optimization. SEMCCO, Part 1. 2011: 127-134.
19. Marjalili S, Mirjalili SM, Lewis A, Grey Wolf Optimizer. Advances in Engineering Software. 2014: 46-61.
